# Sine-G family of distributions in Bayesian survival modeling: A baseline hazard approach for proportional hazard regression with application to right-censored oncology datasets using R and STAN

**DOI:** 10.1371/journal.pone.0307410

**Published:** 2025-03-13

**Authors:** Abdisalam Hassan Muse, Amani Almohaimeed, Hana N. Alqifari, Christophe Chesneau

**Affiliations:** 1 Research and Innovation Centre, Amoud University, Amoud valley, Borama, Somalia; 2 Department of Statistics and Operations Research, College of Science, Qassim University, Buraydah, Saudi Arabia; 3 Department of Mathematics, LMNO, CNRS-Université de Caen, Campus II, Caen, France; University of Eastern Finland, FINLAND

## Abstract

In medical research and clinical practice, Bayesian survival modeling is a powerful technique for assessing time-to-event data. It allows for the incorporation of prior knowledge about the model’s parameters and provides a more comprehensive understanding of the underlying hazard rate function. In this paper, we propose a Bayesian survival modeling strategy for proportional hazards regression models that employs the Sine-G family of distributions as baseline hazards. The Sine-G family contains flexible distributions that can capture a wide range of hazard forms, including increasing, decreasing, and bathtub-shaped hazards. In order to capture the underlying hazard rate function, we examine the flexibility and effectiveness of several distributions within the Sine-G family, such as the Gompertz, Lomax, Weibull, and exponentiated exponential distributions. The proposed approach is implemented using the R programming language and the STAN probabilistic programming framework. To evaluate the proposed approach, we use a right-censored survival dataset of gastric cancer patients, which allows for precise determination of the hazard rate function while accounting for censoring. The Watanabe Akaike information criterion and the leave-one-out information criterion are employed to evaluate the performance of various baseline hazards.

## 1 Introduction

### 1.1 Background of the study

In survival analysis, it is important to accurately model the underlying hazard rate function (HRF) in order to estimate individual survival probabilities and arrive at informed treatment decisions. Nevertheless, the complex structure of real-world survival data is often challenging to accurately represent with traditional survival analysis techniques. A common option for modeling survival data is the proportional hazard (PH) regression model, which enables the addition of factors that might have an impact on the HRF. According to [1, 2], the PH model’s popularity comes from its simplicity in handling technical problems like censoring and truncation, which are caused by the HRF’s appealing interpretation as a risk that varies with time. The baseline HRF, according to the traditional PH model, is assumed to follow a certain distribution, such as the Weibull, Exponential, or Gompertz distribution.

On the other hand, Bayesian survival modeling is an effective method for predicting the time to an event such as death, illness development, or failure in the system. It produces a more accurate model and meaningful conclusions by allowing the model to incorporate uncertainty and prior knowledge. [3] pointed out that using classical models to perform survival analysis may result in inaccurate conclusions when there are insufficient events or when the effective sample size is small; in such cases, Bayesian analysis can yield more reliable results.

In recent years, distribution theory has been one of the most active branches of probability and statistics. In particular, researchers have proposed several forms of statistical distributions based on trigonometrical transformation to model data and thus discover the best-suited distribution in statistics theory and practice. [4] reviewed and compared the latest improvements and contributions made through the various families of trigonometric functions in distribution and their application in data modeling, as well as their properties. As a matter of fact, the main trigonometric family is the sine-generated (Sine-G) family introduced in [5], which demonstrates how the sine function can be used quite efficiently to modify or enhance the modeling power of a baseline distribution. More investigations have been conducted in this direction. For instance, [6] proposed a new family based on a sine transformation known as the new Sine-G family, which has been demonstrated to have significant features. Furthermore, [7] demonstrated that the transformed sine-Weibull (TSW) distribution, which belongs to the Sine-G family, holds great potential for lifetime data analysis and modeling. Using a composition scheme, [8] introduced another extension of the Sine-G family called the Sine Kumaraswamy-G family and demonstrated its potential and robustness. The exponentiated Sine-G family was proposed by [9] and has also important properties for lifetime studies. By combining a trigonometric function with the Marshall-Olkin approach, [10] developed the Marshall-Olkin Sine-G (MOS-G) family.

By investigating the use of trigonometric distributions, specifically the Sine-G family, as baseline hazards for PH regression models, this study aims to introduce a new approach in survival modeling. Trigonometric distributions have specific properties that make them useful for modeling complex HRFs in challenging scenarios, such as right-censored oncology datasets. The robustness of the Bayesian technique combined with the flexibility and performance of the Sine-G family will also improve decision-making, survival predictions, and the study of medical research and clinical practice.

The effectiveness of statistical models, particularly survival models, is frequently evaluated using the Watanabe-Akaike information criterion (WAIC) [11] and the leave-one-out information criterion (LOOIC) [12]. WAIC and LOOIC are both completely Bayesian information criteria. Therefore, they consider the full posterior distribution of model parameters. As a result, they are more reliable than other information criteria that rely on asymptotic approximations, including the Bayesian information criterion (BIC), deviance information criterion (DIC) and Akaike information criterion (AIC). See [13, 14] for further information. In the context of the Sine-G family of survival distributions, WAIC and LOOIC can be used to assess the performance of different baseline hazards. The hazard rate for a standard individual, or an individual without any variables, is known as the baseline hazard. To find the baseline hazard that most accurately predicts the data and best fits the data, we can take into account the WAIC and LOOIC. The best-fitting model would be the one with the lowest WAIC or LOOIC value.

### 1.2 Motivation statement

The motivation for this research paper stems from the limitations of traditional survival analysis techniques in capturing complex hazard rate patterns exhibited in real-world datasets. Existing approaches, such as the use of exponential, Weibull, Lomax, and Gompertz distributions as baseline hazards in proportional hazards (PH) regression, often fail to adequately model non-monotonic hazard rates, including unimodal and bathtub shapes [15–21].

To address this issue, we propose a novel solution by introducing the Sine-G family of distributions as baseline hazards within a Bayesian survival modeling framework. The main motivation behind this approach is the inherent flexibility of the Sine-G family, which can capture a wider range of hazard rate patterns without the need for additional parameters. Our proposed models are capable of accurately modeling both monotonic and non-monotonic hazard rates without adding extra parameters to the baseline distribution or complicating the estimation of the parameters.

By incorporating these trigonometric distributions into survival regression models, we aim to provide a more accurate and comprehensive representation of survival times, particularly in right-censored gastric oncology datasets. The benefits of our proposed models are twofold. Firstly, they offer a more flexible and robust method for capturing complex hazard rate patterns, allowing researchers and practitioners to accurately model a wider range of real-world scenarios. Secondly, our models achieve this enhanced flexibility without the need for additional parameters, simplifying the estimation process and ensuring ease of implementation.

Our research contributes to advancing the field of survival analysis by enhancing the modeling options available for researchers and practitioners. By addressing the limitations of traditional approaches and offering a more comprehensive representation of survival times, our proposed models lead to improved insights and predictions in the study of time-to-event outcomes. Furthermore, the simplicity and effectiveness of our approach make it a valuable tool for various applications in the medical field and beyond.

### 1.3 Novelty of the study

To the best of the authors’ knowledge, this paper represents the first of its kind in incorporating trigonometric families of distributions as baseline hazards within parametric survival regression models. This novel approach challenges the conventional assumptions and opens up new avenues for understanding the underlying relationships in survival data. Furthermore, this study also pioneers the utilization of a Bayesian approach, specifically using the Stan language, to estimate the parameters and conduct inference in the proposed models. By leveraging the flexibility of Bayesian methods, we can account for uncertainty, incorporate prior knowledge, and obtain more robust and interpretable results.

### 1.4 Main contributions

The main contributions of this paper can be summarized as follows:

- Introducing the Sine-G family of distributions as baseline hazards: By considering trigonometric families of distributions, we provide a new perspective for modeling survival data, potentially capturing complex patterns that may not be adequately represented by traditional distributions.
- Developing a baseline hazard approach for PH regression models: We present a methodology to incorporate the Sine-G family of distributions as baseline hazards within parametric survival regression models, allowing for more flexible and tailored modeling of survival times.
- Proposing a Bayesian framework using Stan: We demonstrate the applicability of a Bayesian approach for parameter estimation and inference in the proposed models. The use of Stan language enables efficient computation, uncertainty quantification, and the integration of prior knowledge.
- Application to right-censored gastric oncology datasets: We apply the proposed methodology to real-world gastric oncology datasets with right-censored observations, showcasing its effectiveness in capturing the underlying survival patterns and providing valuable insights for clinical research and decision-making.

Overall, this research contributes to the advancement of survival analysis by introducing novel modeling techniques, leveraging Bayesian inference, and providing a comprehensive analysis of survival times in the context of gastric oncology.

### 1.5 Outline of the paper

This paper presents a comprehensive study on Bayesian survival modeling using the Sine-G family of distributions as baseline hazards for PH regression models. Section 2 discusses the functional basis of the Sine-G family, the different baseline hazards considered, and the PH regression model. The Bayesian methodology for the proposed framework is presented in Section 3. Using a lung cancer right-censored survival dataset, we assess the effectiveness of different baseline hazards for the Sine-G family in Section 4. To select the best-fitting distribution for capturing the dataset’s complex survival dynamics, we apply WAIC and LOOIC. Section 5 summarizes the findings, comparison, and evaluation of the different baseline hazards. Finally, in Sections 6 and 7, we present conclusions, recommendations, and future study areas, underscoring the value of the proposed approach in improving survival predictions and developing decision-making in medical research and clinical practice.

## 2 Sine-G distributions family

### 2.1 Main functions

Modeling a wide variety of HRFs is possible with the flexible distributions of the Sine-G family. This was shown in [5, 8, 22], and the related references mentioned in the introductory section. With respect to time *t*, let us now define the various possible structures using the HRF, survival function (SF), cumulative HRF (CHRF), and odds function. To begin, the CDF associated with the Sine-G family is indicated as

$$\begin{array}{c} F_{sin}\left(t;\theta \right)=\int_{0}^{G(t;\theta )}\frac{\pi }{2}\;\text{cos}(\frac{\pi }{2}x)dx, \end{array}$$ (1)

which is the integral of a basic cosine probability density function (PDF), upon composition with a CDF of the baseline distribution denoted *G*(*t*; *θ*). The “*θ*” symbolizes all the parameters on which the baseline distribution depends; it can be considered a vector in the article. After a simple integration, Eq (1) can be written as

$$\begin{array}{c} F_{\text{sin}}\left(t;\theta \right)=\text{sin}(\frac{\pi }{2}G\left(t;\theta \right)). \end{array}$$ (2)

If the PDF associated with *G*(*t*; *θ*) is denoted as *g*(*t*; *θ*), the PDF of the family is given by

$$\begin{array}{c} f_{\text{sin}}\left(t;\theta \right)=\frac{\pi }{2}g\left(t;\theta \right)\;\text{cos}(\frac{\pi }{2}G\left(t;\theta \right)). \end{array}$$ (3)

Directly, the SF is given by

$$\begin{array}{c} S_{\text{sin}}\left(t;\theta \right)=1-\text{sin}(\frac{\pi }{2}G\left(t;\theta \right)). \end{array}$$ (4)

Based on the SF and PDF, the HRF is specified by the following ratio function:

$$\begin{array}{c} h_{\text{sin}}\left(t;\theta \right)=\frac{f_{\text{sin}}\left(t;\theta \right)}{S_{\text{sin}}\left(t;\theta \right)}=\frac{\frac{\pi }{2}g\left(t;\theta \right)\;\text{cos}(\frac{\pi }{2}G\left(t;\theta \right))}{1-\text{sin}(\frac{\pi }{2}G\left(t;\theta \right))}. \end{array}$$ (5)

The CHRF is given by

$$\begin{array}{c} H_{\text{sin}}\left(t;\theta \right)=-\text{log}\{S_{\text{sin}\;}\left(t;\theta \right)\}=-\text{log}\{1-\text{sin}(\frac{\pi }{2}G\left(t;\theta \right))\}. \end{array}$$ (6)

To end this part, the odds function is given by the following ratio function:

$$\begin{array}{c} R_{\text{sin}}\left(t;\theta \right)=\frac{F_{\text{sin}}\left(t;\theta \right)}{S_{\text{sin}}\left(t;\theta \right)}=\frac{\;\text{sin}(\frac{\pi }{2}G\left(t;\theta \right))}{1-\text{sin}(\frac{\pi }{2}G\left(t;\theta \right))}. \end{array}$$ (7)

Several baseline distributions will be considered in our findings, and the associated sine distribution will be investigated.

### 2.2 Exponential baseline distribution

To begin, suppose the duration of survival, say modeled by a random variable *T* to fix the notation, is governed by the exponential distribution characterized by a scale parameter (λ). In this context, the CDF and PDF of the exponential distribution can be expressed as

$$\begin{array}{c} G\left(t;\lambda \right)=P\left(T\leq t\right)=1-e^{-\lambda t};\;t\geq 0 \end{array}$$ (8)

and

$$\begin{array}{c} g\left(t;\lambda \right)=\lambda e^{-\lambda t};\;t\geq 0, \end{array}$$ (9)

respectively. It is understood that *G*(*t*;λ) = *g*(*t*;λ) = 0 for *t* < 0. We will omit such complementary value functions in the rest of the study for the sake of simplicity in exposition.

The sine-exponential (SE) distribution is derived by incorporating the exponential distribution as the baseline distribution, defined by *G*(*t*; *θ*), into the Sine-G family. Consequently, the CDF, PDF, SF, CHRF, HRF, and odds function of the SE distribution can be formulated as follows:

$$\begin{array}{c} F\left(t;\lambda \right)=\text{sin}(\frac{\pi }{2}\left(1-e^{-\lambda t}\right));\;t\geq 0, \end{array}$$ (10)

$$\begin{array}{c} f\left(t;\lambda \right)=\frac{\pi }{2}\lambda e^{-\lambda t}\;\text{cos}(\frac{\pi }{2}\left(1-e^{-\lambda t}\right));\;t\geq 0, \end{array}$$ (11)

$$\begin{array}{c} S\left(t;\lambda \right)=1-\text{sin}(\frac{\pi }{2}\left(1-e^{-\lambda t}\right));\;t\geq 0, \end{array}$$ (12)

$$\begin{array}{c} H\left(t;\lambda \right)=-\text{log}\{1-\text{sin}(\frac{\pi }{2}\left(1-e^{-\lambda t}\right))\};\;t\geq 0, \end{array}$$ (13)

$$\begin{array}{c} h\left(t;\lambda \right)=\frac{\frac{\pi }{2}\lambda e^{-\lambda t}\;\text{cos}(\frac{\pi }{2}\left(1-e^{-\lambda t}\right))}{1-\text{sin}(\frac{\pi }{2}\left(1-e^{-\lambda t}\right))};\;t\geq 0 \end{array}$$ (14)

and

$$\begin{array}{c} R\left(t;\lambda \right)=\frac{\;\text{sin}(\frac{\pi }{2}\left(1-e^{-\lambda t}\right))}{1-\text{sin}(\frac{\pi }{2}\left(1-e^{-\lambda t}\right))};\;t\geq 0, \end{array}$$ (15)

respectively.

### 2.3 Weibull baseline distribution

We now consider a more general scenario than the exponential baseline distribution. Suppose the duration of survival is governed by the Weibull distribution characterized by two parameters: a scale parameter (λ) and a shape parameter (*α*). In this context, the CDF, PDF, and HRF of the Weibull distribution can be written as follows:

$$\begin{array}{c} G\left(t;\lambda ,\alpha \right)=1-e^{-{\left(\lambda t\right)}^{\alpha }};\;t\geq 0 \end{array}$$ (16)

and

$$\begin{array}{c} g\left(t;\lambda ,\alpha \right)=\lambda \alpha {\left(\lambda t\right)}^{\alpha -1}e^{-{\left(\lambda t\right)}^{\alpha }};\;t\geq 0, \end{array}$$ (17)

respectively.

In a similar way than the SE distribution, the sine-Weibull (SW) distribution is derived by incorporating the Weibull distribution as the baseline distribution, defined by *G*(*t*; *θ*), into the Sine-G family. Consequently, the CDF, PDF, SF, CHRF, HRF, and odds function of the SW distribution can be specified as follows:

$$\begin{array}{c} F\left(t;\lambda ,\alpha \right)=\text{sin}(\frac{\pi }{2}\left(1-e^{-{\left(\lambda t\right)}^{\alpha }}\right));\;t\geq 0, \end{array}$$ (18)

$$\begin{array}{c} f\left(t;\lambda ,\alpha \right)=\frac{\pi }{2}\lambda \alpha {\left(\lambda t\right)}^{\alpha -1}e^{-{\left(\lambda t\right)}^{\alpha }}\;\text{cos}(\frac{\pi }{2}\left(1-e^{-{\left(\lambda t\right)}^{\alpha }}\right));\;t\geq 0, \end{array}$$ (19)

$$\begin{array}{c} S\left(t;\lambda ,\alpha \right)=1-\text{sin}(\frac{\pi }{2}\left(1-e^{-{\left(\lambda t\right)}^{\alpha }}\right));\;t\geq 0, \end{array}$$ (20)

$$\begin{array}{c} H\left(t;\lambda ,\alpha \right)=-\text{log}\{1-\text{sin}(\frac{\pi }{2}\left(1-e^{-{\left(\lambda t\right)}^{\alpha }}\right))\};\;t\geq 0, \end{array}$$ (21)

$$\begin{array}{c} h\left(t;\lambda ,\alpha \right)=\frac{\frac{\pi }{2}\lambda \alpha {\left(\lambda t\right)}^{\alpha -1}e^{-{\left(\lambda t\right)}^{\alpha }}\;\text{cos}(\frac{\pi }{2}\left(1-e^{-{\left(\lambda t\right)}^{\alpha }}\right))}{1-\text{sin}(\frac{\pi }{2}\left(1-e^{-{\left(\lambda t\right)}^{\alpha }}\right))};\;t\geq 0 \end{array}$$ (22)

and

$$\begin{array}{c} R\left(t;\lambda ,\alpha \right)=\frac{\;\text{sin}(\frac{\pi }{2}\left(1-e^{-{\left(\lambda t\right)}^{\alpha }}\right))}{1-\text{sin}(\frac{\pi }{2}\left(1-e^{-{\left(\lambda t\right)}^{\alpha }}\right))};\;t\geq 0, \end{array}$$ (23)

respectively.

### 2.4 Lomax baseline distribution

A polynomial decay baseline distribution is now considered. Suppose the duration of survival is governed by the Lomax distribution characterized by two parameters: a scale parameter (λ) and a shape parameter (*α*). In this context, the CDF and PDF of the Lomax distribution can be expressed as follows:

$$\begin{array}{c} G\left(t;\lambda ,\alpha \right)=1-{\left(1+\lambda t\right)}^{-\alpha };\;t\geq 0 \end{array}$$ (24)

and

$$\begin{array}{c} g\left(t;\lambda ,\alpha \right)=\alpha \lambda {\left(1+\lambda t\right)}^{-(\alpha +1)};\;t\geq 0, \end{array}$$ (25)

respectively.

As for the previous sine-generated distributions, the sine-Lomax (SL) distribution is derived by incorporating the Lomax distribution as the baseline distribution, defined by *G*(*t*; *θ*), into the Sine-G family. Consequently, the CDF, PDF, SF, CHRF, HRF, and odds function of the SL distribution can be indicated as follows:

$$\begin{array}{c} F\left(t;\alpha ,\beta \right)=\text{sin}(\frac{\pi }{2}\left(1-{\left(1+\lambda t\right)}^{-\alpha }\right));\;t\geq 0, \end{array}$$ (26)

$$\begin{array}{c} f\left(t;\lambda ,\alpha \right)=\frac{\pi }{2}\alpha \lambda {\left(1+\lambda t\right)}^{-(\alpha +1)}\;\text{cos}(\frac{\pi }{2}\left(1-{\left(1+\lambda t\right)}^{-\alpha }\right));\;t\geq 0, \end{array}$$ (27)

$$\begin{array}{c} S\left(t;\lambda ,\alpha \right)=1-\text{sin}(\frac{\pi }{2}\left(1-{\left(1+\lambda t\right)}^{-\alpha }\right));\;t\geq 0, \end{array}$$ (28)

$$\begin{array}{c} H\left(t;\lambda ,\alpha \right)=-\text{log}(1-\text{sin}(\frac{\pi }{2}\left(1-{\left(1+\lambda t\right)}^{-\alpha }\right)));\;t\geq 0, \end{array}$$ (29)

$$\begin{array}{c} h\left(t;\lambda ,\alpha \right)=\frac{\frac{\pi }{2}\alpha \lambda {\left(1+\lambda t\right)}^{-(\alpha +1)}\;\text{cos}(\frac{\pi }{2}\left(1-{\left(1+\lambda t\right)}^{-\alpha }\right))}{1-\text{sin}(\frac{\pi }{2}\left(1-{\left(1+\lambda t\right)}^{-\alpha }\right))};\;t\geq 0 \end{array}$$ (30)

and

$$\begin{array}{c} R\left(t;\lambda ,\alpha \right)=\frac{\;\text{sin}(\frac{\pi }{2}\left(1-{\left(1+\lambda t\right)}^{-\alpha }\right))}{1-\text{sin}(\frac{\pi }{2}\left(1-{\left(1+\lambda t\right)}^{-\alpha }\right))};\;t\geq 0, \end{array}$$ (31)

respectively.

### 2.5 Exponentiated exponential baseline distribution

We now examine another extension of the exponential distribution. Suppose the duration of survival is governed by the exponentiated exponential distribution characterized by two parameters: a scale parameter (λ) and a shape parameter (*α*). In this context, the CDF and PDF of the exponentiated exponential distribution can be expressed as follows:

$$\begin{array}{c} G\left(t;\lambda ,\alpha \right)={\left(1-e^{-\lambda t}\right)}^{\alpha };\;t\geq 0 \end{array}$$ (32)

and

$$\begin{array}{c} g\left(t;\lambda ,\alpha \right)=\alpha \lambda e^{-\lambda t}{\left(1-e^{-\lambda t}\right)}^{\alpha -1};\;t\geq 0, \end{array}$$ (33)

respectively.

The sine-exponentiated exponential (SEE) distribution is derived by incorporating the exponentiated exponential distribution as the baseline distribution, defined by *G*(*t*; *θ*), into the Sine-G family. Consequently, the CDF, PDF, SF, CHRF, HRF, and odds function of the SEE distribution can be written as follows:

$$\begin{array}{c} F\left(t;\lambda ,\alpha \right)=\text{sin}(\frac{\pi }{2}{\left(1-e^{-\lambda t}\right)}^{\alpha });\;t\geq 0, \end{array}$$ (34)

$$\begin{array}{c} f\left(t;\lambda ,\alpha \right)=\frac{\pi }{2}\alpha \lambda e^{-\lambda t}{\left(1-e^{-\lambda t}\right)}^{\alpha -1}\;\text{cos}(\frac{\pi }{2}{\left(1-e^{-\lambda t}\right)}^{\alpha });\;t\geq 0, \end{array}$$ (35)

$$\begin{array}{c} S\left(t;\lambda ,\alpha \right)=1-\text{sin}(\frac{\pi }{2}{\left(1-e^{-\lambda t}\right)}^{\alpha });\;t\geq 0, \end{array}$$ (36)

$$\begin{array}{c} H\left(t;\lambda ,\alpha \right)=-\text{log}\{1-\text{sin}(\frac{\pi }{2}{\left(1-e^{-\lambda t}\right)}^{\alpha })\};\;t\geq 0, \end{array}$$ (37)

$$\begin{array}{c} h\left(t;\lambda ,\alpha \right)=\frac{\frac{\pi }{2}\alpha \lambda e^{-\lambda t}{\left(1-e^{-\lambda t}\right)}^{\alpha -1}\;\text{cos}(\frac{\pi }{2}{\left(1-e^{-\lambda t}\right)}^{\alpha })}{1-\text{sin}(\frac{\pi }{2}{\left(1-e^{-\lambda t}\right)}^{\alpha })};\;t\geq 0 \end{array}$$ (38)

and

$$\begin{array}{c} R\left(t;\lambda ,\alpha \right)=\frac{\;\text{sin}(\frac{\pi }{2}{\left(1-e^{-\lambda t}\right)}^{\alpha })}{1-\text{sin}(\frac{\pi }{2}{\left(1-e^{-\lambda t}\right)}^{\alpha })};\;t\geq 0, \end{array}$$ (39)

respectively.

### 2.6 Gompertz baseline distribution

Another baseline is now considered, based on the Gompertz distribution. Thus, suppose the duration of survival is governed by this distribution characterized by two parameters: a scale parameter (λ) and a shape parameter (*α*). In this setting, the CDF, PDF, and HRF of the Gompertz distribution can be written as follows:

$$\begin{array}{c} G\left(t;\lambda ,\alpha \right)=1-\text{exp}(-\alpha \left(e^{\lambda t}-1\right));\;t\geq 0 \end{array}$$ (40)

and

$$\begin{array}{c} g\left(t;\lambda ,\alpha \right)=\lambda \alpha \text{exp}\left(\alpha +\lambda t-\alpha e^{\lambda t}\right);\;t\geq 0, \end{array}$$ (41)

respectively.

The sine-Gompertz (SG) distribution is derived by incorporating the Gompertz distribution as the baseline distribution, defined by *G*(*t*; *θ*), into the Sine-G family. Consequently, the CDF, PDF, SF, CHRF, HRF, and odds function of the SG distribution can be expressed as follows:

$$\begin{array}{c} F\left(t;\lambda ,\alpha \right)=\text{sin}(\frac{\pi }{2}\left(1-\text{exp}\left(-\alpha \left(e^{\lambda t}-1\right)\right)\right));\;t\geq 0, \end{array}$$ (42)

$$\begin{array}{c} f\left(t;\lambda ,\alpha \right)=\frac{\pi }{2}\lambda \alpha \;\text{exp}\left(\alpha +\lambda t-\alpha e^{\lambda t}\right)\;\text{cos}(\frac{\pi }{2}\left(1-\text{exp}\left(-\alpha \left(e^{\lambda t}-1\right)\right)\right));\;t\geq 0, \end{array}$$ (43)

$$\begin{array}{c} S\left(t;\lambda ,\alpha \right)=1-\text{sin}(\frac{\pi }{2}\left(1-\text{exp}\left(-\alpha \left(e^{\lambda t}-1\right)\right)\right));\;t\geq 0, \end{array}$$ (44)

$$\begin{array}{c} H\left(t;\lambda ,\alpha \right)=-\text{log}\{1-\text{sin}(\frac{\pi }{2}\left(1-\text{exp}\left(-\alpha \left(e^{\lambda t}-1\right)\right)\right))\};\;t\geq 0, \end{array}$$ (45)

$$\begin{array}{c} h\left(t;\lambda ,\alpha \right)=\frac{\frac{\pi }{2}\lambda \alpha \;\text{exp}\left(\alpha +\lambda t-\alpha e^{\lambda t}\right)\;\text{cos}(\frac{\pi }{2}\left(1-\text{exp}\left(-\alpha \left(e^{\lambda t}-1\right)\right)\right))}{1-\text{sin}(\frac{\pi }{2}\left(1-\text{exp}\left(-\alpha \left(e^{\lambda t}-1\right)\right)\right))};\;t\geq 0 \end{array}$$ (46)

and

$$\begin{array}{c} R\left(t;\lambda ,\alpha \right)=\frac{\;\text{sin}(\frac{\pi }{2}\left(1-\text{exp}\left(-\alpha \left(e^{\lambda t}-1\right)\right)\right))}{1-\text{sin}(\frac{\pi }{2}\left(1-\text{exp}\left(-\alpha \left(e^{\lambda t}-1\right)\right)\right))};\;t\geq 0, \end{array}$$ (47)

respectively.

### 2.7 Shapes of HRF for one of the proposed baseline distribution

**Fig 1:**
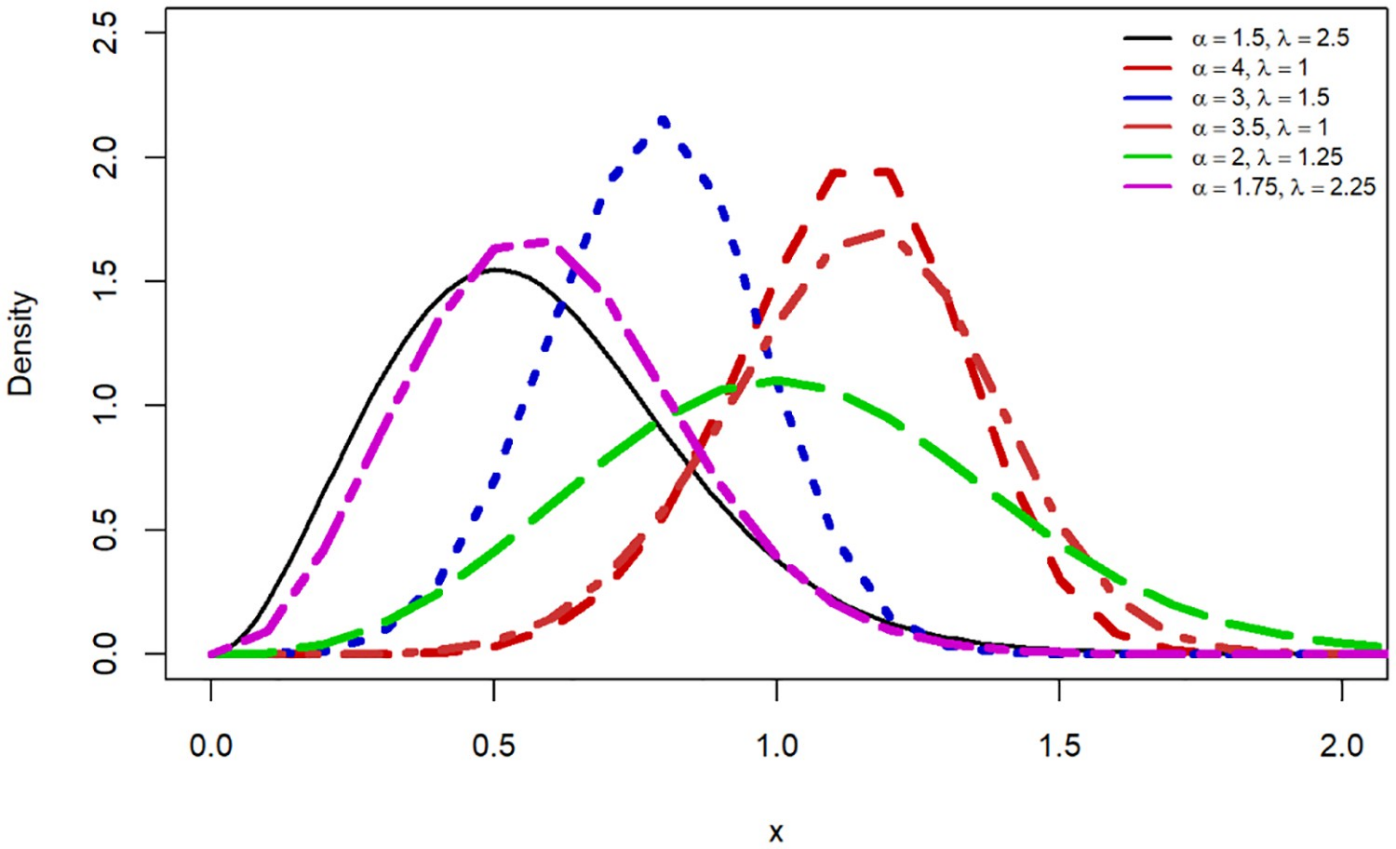
PDF shapes for the SW baseline distribution.

**Fig 2:**
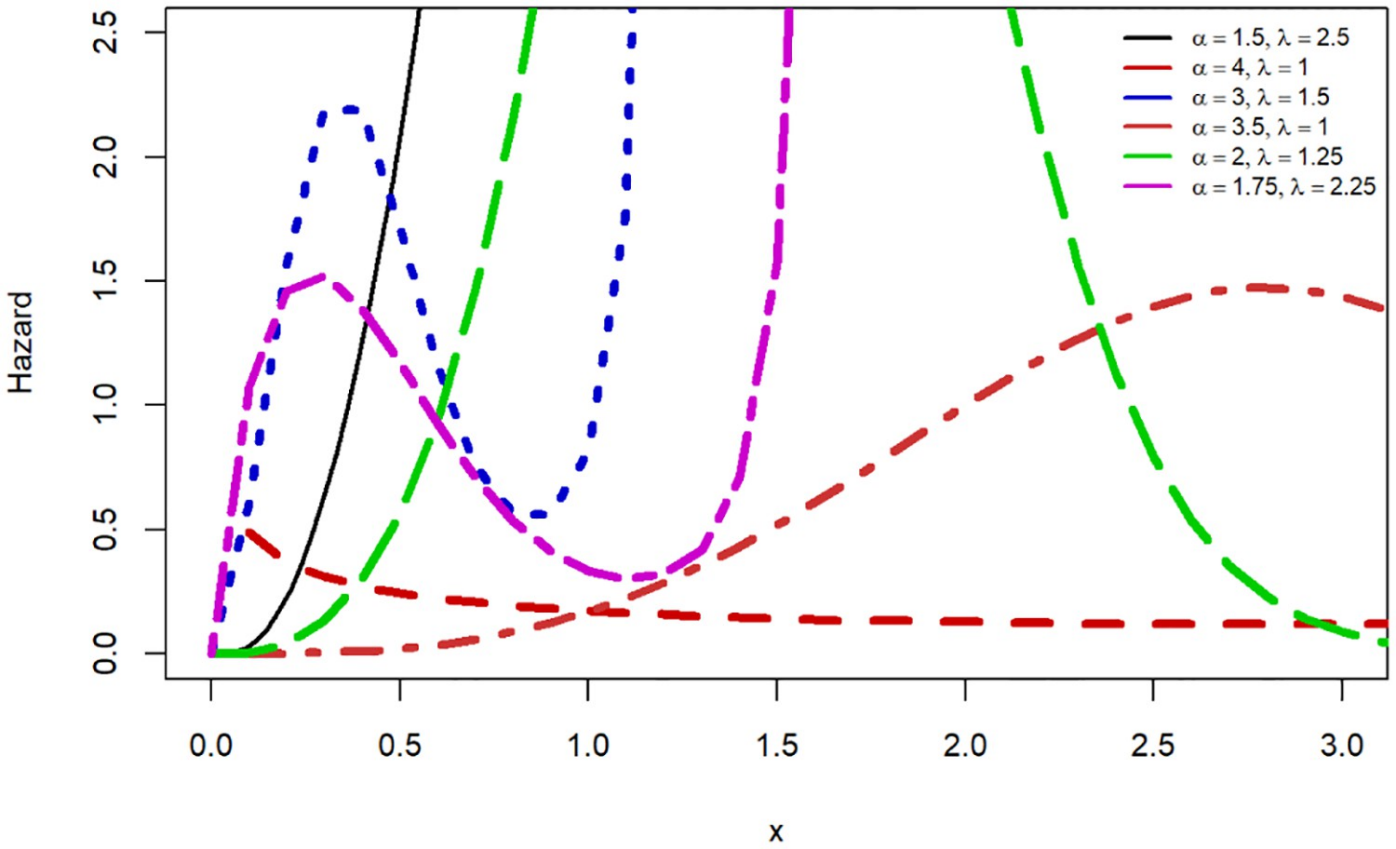
HRF shapes for the SW baseline distribution.

**Fig 3:**
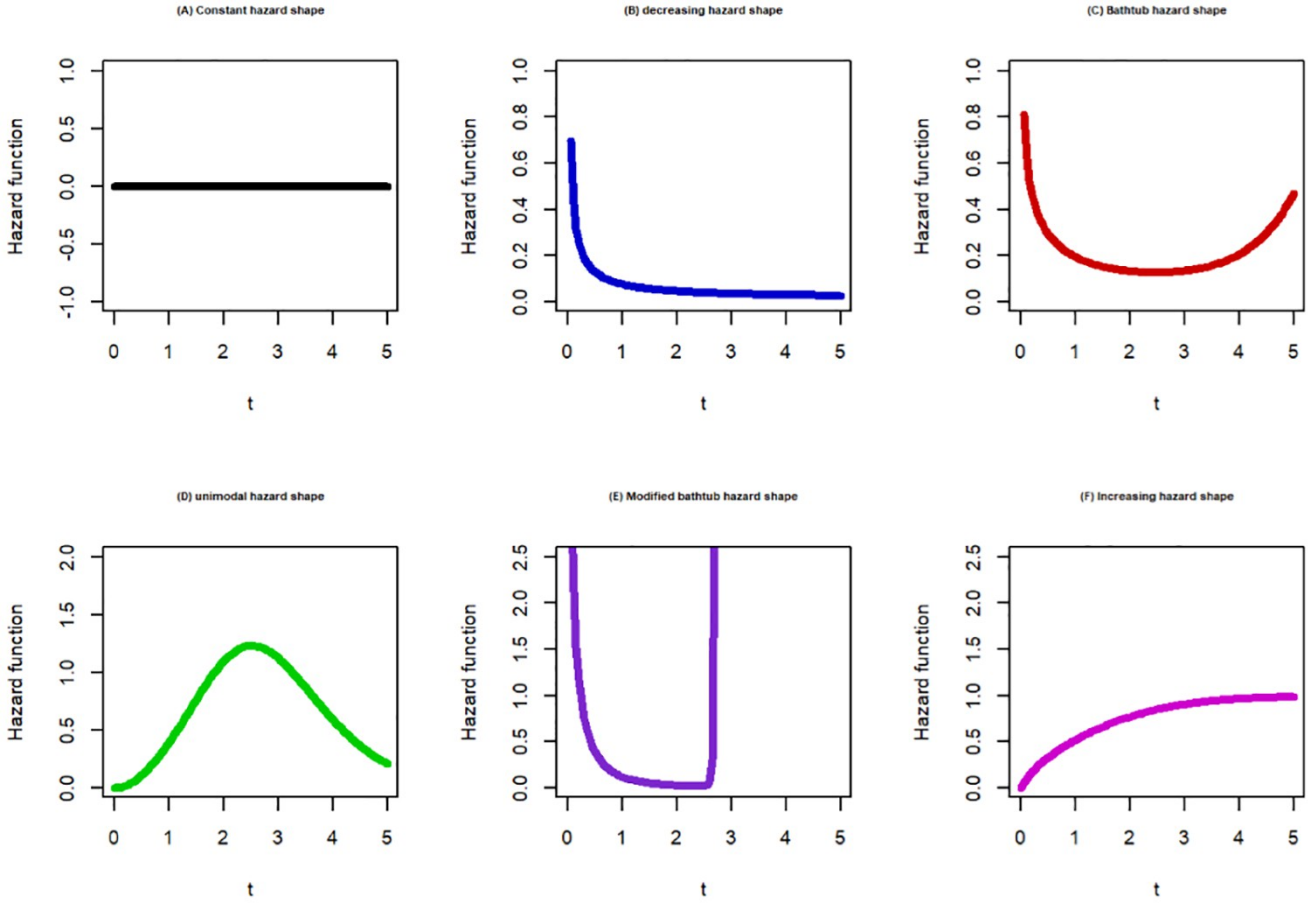
Six patterns of the hrf shapes for the SW baseline distribution.

In this sub-section, we showcased various shapes for probability density function (PDF) as shown in Fig 1, and HRF shape as shown in Fig 2. The HRF shapes encompass six distinct HRF patterns, namely constant, increasing, decreasing, unimodal, bathtub, and modified bathtub shapes as presented in Fig 3.

## 3 PH model

We are now in a position to introduce the proposed PH model and also those derived from the above sine-generated distributions.

### 3.1 Formulation

The Cox PH model, which is based on hazard rates and was introduced by Cox in 1972 [23], is a widely recognized regression model in survival analysis [24–27]. In this model, the HRF is multiplicatively influenced by covariates. Various researchers have conducted studies on the parametric PH model using different baseline distributions and inferential techniques. Rezaei et al. [28] developed and evaluated an extended exponential geometric baseline distribution for the parametric PH model. Khan and Khosa [15] introduced a parametric PH model with a generalized log-logistic (GLL) baseline distribution. Balakrishnan et al. [29] developed an extension of the PH model and a reversed PH model using the Marshall-Olkin baseline hazard. Muse et al. [30] investigated the Bayesian approaches of the PH model with a GLL baseline hazard.

The PH regression model’s HRF, SF, and the CHRF can be expressed as follows:

$$\begin{array}{c} h_{PH}\left(t;\theta ,\beta ,x\right)=h_{0}\left(t;\theta \right)e^{x^{\prime }\beta };\;t\geq 0, \end{array}$$ (48)

$$\begin{array}{c} S_{PH}\left(t;\theta ,\beta ,x\right)=S_{0}{\left(t;\theta \right)}^{e^{x^{\prime }\beta }};\;t\geq 0 \end{array}$$ (49)

and

$$\begin{array}{c} H_{PH}\left(t;\theta ,\beta ,x\right)=H_{0}\left(t;\theta \right)e^{x^{\prime }\beta };\;t\geq 0, \end{array}$$ (50)

where *h*_0_(*t*; *θ*), *S*_0_(*t*; *θ*), and *H*_0_(*t*; *θ*) are the baseline hazard rate, survival and cumulative hazard rate functions, *θ* symbolizes all the parameters on which the baseline hazard depends, *x*′ = (*x*_1_, *x*_2_, …, *x*_*n*_) denotes the vector of the covariates in the survival model and *β* is the vector of unknown regression coefficients, excluding an intercept term to ensure model identifiability.

#### 3.1.1 Likelihood function for the PH model

Assuming we possess a random sample of size *n* and we observe (*t*_*i*_, *δ*_*i*_, *x*_*i*_), *i* = 1, …, *n*, where *t*_*i*_ represents the observed lifetime for the *i*-th individual, *δ*_*i*_ denotes the censoring status (1 if the event of interest has occurred, 0 otherwise), and *x*_*i*_ represents the explanatory variables. Regarding the PH model (48), the likelihood function can be expressed as follows:

$$\begin{array}{c} \begin{array}{cc} L(\theta ,\beta ;D) & =\prod_{i=1}^{n}{\left[f(t_{i};\theta ,\beta ,x_{i})\right]}^{\delta_{i}}{\left[S(t_{i};\theta ,\beta ,x_{i})\right]}^{1-\delta_{i}}\\ & =\prod_{i=1}^{n}{\left[\frac{h(t_{i};\theta ,\beta ,x_{i})}{S(t_{i};\theta ,\beta ,x_{i})}\right]}^{\delta_{i}}{\left[S(t_{i};\theta ,\beta ,x_{i})\right]}^{1-\delta_{i}}\\ & =\prod_{i=1}^{n}{\left[h(t_{i};\theta ,\beta ,x_{i})\right]}^{\delta_{i}}S(t_{i};\theta ,\beta ,x_{i})\\ & =\prod_{i=1}^{n}{\left[h(t_{i};\theta ,\beta ,x_{i})\right]}^{\delta_{i}}\;\text{exp}[-H(t_{i};\theta ,\beta ,x_{i})]\\ & =\prod_{i=1}^{n}{\left[h_{0}(t_{i};\theta )e^{x_{i}^{\prime }\beta }\right]}^{\delta_{i}}\;\text{exp}[-H_{0}(t_{i};\theta )e^{x_{i}^{\prime }\beta }], \end{array} \end{array}$$ (51)

where *D* = (*t*_*i*_, *δ*_*i*_, *x*_*i*_, *i* = 1, 2, …, *n*), which can be described as a matrix with dimensions (3 × *n*), where each column represents the specific components of the event data for individual *i*. The first row of *D* corresponds to the observed time *t*_*i*_, the second row represents the event indicator *δ*_*i*_ (with 1 indicating an event occurrence and 0 for censored data), and the third row denotes the set of covariates or explanatory variables *x*_*i*_ associated with the individual. More precisely, we can write

$$\begin{array}{c} D=[\begin{array}{cccc} t_{1} & t_{2} & \ldots & t_{n}\\ \delta_{1} & \delta_{2} & \ldots & \delta_{n}\\ x_{1} & x_{2} & \ldots & x_{n} \end{array}]. \end{array}$$

The natural logarithm of the likelihood function, commonly known as the log-likelihood function, is given by

$$\begin{array}{c} \begin{array}{cc} \ell (\theta ,\beta ;D)= & \text{log}\;L\left(\theta ,\beta ;D\right)=\sum_{i=1}^{n}\delta_{i}\;\text{log}[h_{0}(t_{i};\theta )+x_{i}^{\prime }\beta ]-\sum_{i=1}^{n}H_{0}(t_{i};\theta )e^{x_{i}^{\prime }\beta }. \end{array} \end{array}$$ (52)

In this equation, we recall that *h*_0_(*t*; *θ*) and *H*_0_(*t*; *θ*) represent the baseline HRF and CHRF for the proposed baseline distributions derived from the Sine-G family, respectively. The complete log-likelihood function of the Sin-G PH regression model can be easily expressed using this formulation.

The maximum likelihood estimates (MLEs) of the parameters are determined by maximizing this function. They can be obtained through an iterative optimization process, such as the Newton-Raphson algorithm. The MLEs tend to approach normality, enabling hypothesis testing and interval estimations of model parameters.

#### 3.1.2 Survival data generation from the PH regression models

In our study, we used the inversion approach to generate lifetime data from the PH regression model. This particular technique relies on the connection between the CHRF of a survival random variable and a standard uniform random variable. Whenever the CHRF can be expressed in a closed-form solution, this method can be applied, inverted, and conveniently implemented using the R software [31].

In this study, we adopted the approach proposed by Bender et al. [32] for simulating data from the Cox PH model. Additional details can be found in references [33, 34].

The CDF is derived from the survival function using the following general equation:

$$\begin{array}{c} F(t;x)=1-S(t;x). \end{array}$$ (53)

where *t* represents the survival time and *x* denotes the covariates. Consequently, if *Y* is a random variable following the CDF *F*, then *U* = *F*(*Y*) follows a uniform distribution on the interval [0, 1], and (1 − *U*) also follows the same distribution. It can be observed that

$$\begin{array}{c} 1-U=S\left(T\right)=\text{exp}\{-H_{0}\left(T;\theta ,\beta ,x\right)\}, \end{array}$$ (54)

which implies

$$\begin{array}{c} \text{exp}\{-H_{0}\left(T;\theta ,\beta ,x\right)\}=1-U. \end{array}$$ (55)

Considering CHRF for the PH model in Eq (50), we obtain

$$\begin{array}{c} \text{exp}\{-H_{0}(T;\theta )e^{x^{\prime }\beta }\}=1-U. \end{array}$$ (56)

The generation of life times for the proposed PH regression model is as follows:

$$\begin{array}{c} T=H_{0}^{-1}(\frac{-\text{log}(1-U)}{e^{x^{\prime }\beta }};\theta ). \end{array}$$

If the baseline HRF is strictly positive for all *t*, then the baseline CHRF can be inverted, allowing us to express the lifetime data for each of the PH regression models considered in this study.

Here, we used the baseline CHRF and its inverse function, i.e., *H*_0_(*t*; *θ*) and $H_{0}^{-1}\left(t;\theta \right)$, to generate the lifetime data.

### 3.2 Proposed PH models

#### 3.2.1 Sine-Exponential PH model

Assume that a random variable *T* has a SE baseline hazard with parameter λ. Consequently, the HRF and CHRF with covariate variable vector *x* are as follows:

$$\begin{array}{c} h_{SE-PH}\left(t;\lambda ,\beta ,x\right)=\frac{\frac{\pi }{2}\lambda e^{-\lambda t}\;\text{cos}(\frac{\pi }{2}\left(1-e^{-\lambda t}\right))}{1-\text{sin}(\frac{\pi }{2}\left(1-e^{-\lambda t}\right))}e^{x^{\prime }\beta };\;t\geq 0 \end{array}$$ (57)

and

$$\begin{array}{c} H_{SE-PH}\left(t;\lambda ,\beta ,x\right)=-\text{log}\{1-\text{sin}(\frac{\pi }{2}\left(1-e^{-\lambda t}\right))\}e^{x^{\prime }\beta };\;t\geq 0, \end{array}$$ (58)

respectively.

#### 3.2.2 Sine-Weibull PH model

Assume *T* has a SW baseline hazard with parameters λ and *α*. Consequently, the HRF and CHRF with covariate variable vector *x* are as follows:

$$\begin{array}{c} H_{SW-PH}\left(t;\lambda ,\alpha \right)=-\text{log}\{1-\text{sin}(\frac{\pi }{2}\left(1-e^{-{\left(\lambda t\right)}^{\alpha }}\right))\}e^{x^{\prime }\beta };\;t\geq 0 \end{array}$$ (59)

and

$$\begin{array}{c} h_{SW-PH}\left(t;\lambda ,\alpha \right)=\frac{\frac{\pi }{2}\lambda \alpha {\left(\lambda t\right)}^{\alpha -1}e^{-{\left(\lambda t\right)}^{\alpha }}\;\text{cos}(\frac{\pi }{2}\left(1-e^{-{\left(\lambda t\right)}^{\alpha }}\right))}{1-\text{sin}(\frac{\pi }{2}\left(1-e^{-{\left(\lambda t\right)}^{\alpha }}\right))}e^{x^{\prime }\beta };\;t\geq 0, \end{array}$$ (60)

respectively.

#### 3.2.3 Sine-Lomax PH model

Assume *T* has a SL baseline hazard with parameters λ and *α*. Consequently, the HRF and CHRF with covariate variable vector *x* are as follows:

$$\begin{array}{c} H_{SL-PH}\left(t;\lambda ,\alpha \right)=-\text{log}(1-\text{sin}(\frac{\pi }{2}\left(1-{\left(1+\lambda t\right)}^{-\alpha }\right)))e^{x^{\prime }\beta };\;t\geq 0 \end{array}$$ (61)

and

$$\begin{array}{c} h_{SL-PH}\left(t;\lambda ,\alpha \right)=\frac{\frac{\pi }{2}\alpha \lambda {\left(1+\lambda t\right)}^{-(\alpha +1)}\;\text{cos}(\frac{\pi }{2}\left(1-{\left(1+\lambda t\right)}^{-\alpha }\right))}{1-\text{sin}(\frac{\pi }{2}\left(1-{\left(1+\lambda t\right)}^{-\alpha }\right))}e^{x^{\prime }\beta };\;t\geq 0, \end{array}$$ (62)

respectively.

#### 3.2.4 Sine-exponentiated exponential PH model

Assume *T* has a SEE baseline hazard with parameters λ and *α*. Consequently, the HRF and CHRF with covariate variable vector *x* are as follows:

$$\begin{array}{c} H_{SEE-PH}\left(t;\lambda ,\alpha \right)=-\text{log}\{1-\text{sin}(\frac{\pi }{2}{\left(1-e^{-\lambda t}\right)}^{\alpha })\}e^{x^{\prime }\beta };\;t\geq 0 \end{array}$$ (63)

and

$$\begin{array}{c} h_{SEE-PH}\left(t;\lambda ,\alpha \right)=\frac{\frac{\pi }{2}\alpha \lambda e^{-\lambda t}{\left(1-e^{-\lambda t}\right)}^{\alpha -1}\;\text{cos}(\frac{\pi }{2}{\left(1-e^{-\lambda t}\right)}^{\alpha })}{1-\text{sin}(\frac{\pi }{2}{\left(1-e^{-\lambda t}\right)}^{\alpha })}e^{x^{\prime }\beta };\;t\geq 0, \end{array}$$ (64)

respectively.

#### 3.2.5 Sine-Gompertz PH model

Assume *T* has a SG baseline hazard with parameters λ and *α*. Consequently, the HRF and CHRF with covariate variable vector *x* and *t* ≥ 0, are respectively as follows:

$$\begin{array}{c} H_{SG-PH}\left(t;\lambda ,\alpha \right)=-\text{log}\{1-\text{sin}(\frac{\pi }{2}\left(1-\text{exp}\left(-\alpha \left(e^{\lambda t}-1\right)\right)\right))\}e^{x^{\prime }\beta }, \end{array}$$ (65)

and

$$\begin{array}{c} h_{SG-PH}\left(t;\lambda ,\alpha \right)=\frac{\frac{\pi }{2}\lambda \alpha \;\text{exp}\left(\alpha +\lambda t-\alpha e^{\lambda t}\right)cos(\frac{\pi }{2}\left(1-exp\left(-\alpha \left(e^{\lambda t}-1\right)\right)\right))}{1-sin(\frac{\pi }{2}\left(1-exp\left(-\alpha \left(e^{\lambda t}-1\right)\right)\right))}e^{x^{\prime }\beta } \end{array}$$ (66)

## 4 Bayesian analysis

### 4.1 Prior specification

In this subsection, we present general guidelines for selecting priors for regression coefficients associated with explanatory variables and baseline hazard parameters. We examined the prior independence scenario between the baseline parameters in *h*_0_(*t*) (baseline hazard) and the regression coefficients. Furthermore, we assumed prior independence of the regression coefficients in a non-informative setting by using normal distributions with zero mean and a large known variance [2, 35], given by

$$\begin{array}{c} \pi \left(h_{0},\beta \right)=\pi \left(h_{0}\right)\pi \left(\beta \right)=\pi \left(h_{0}\right)\prod_{j=1}^{J}N\left(\beta_{j}|0,\sigma_{j}^{2}\right), \end{array}$$ (67)

where $N(\beta_{j}|0,\sigma_{j}^{2})$ represents a normal distribution with zero mean, variance of $\sigma_{j}^{2}$, and regression coefficient *β*_*j*_ for the *j*-th covariate where *j* = 1, 2, …, *J*, *π*(*h*_0_) signifies the prior distribution of all baseline parameters and hyperparameters in *h*_0_(*t*).

For the baseline hazard parameter *θ* in baseline distributions, say *θ* = (λ, *α*), we employ gamma distributions as prior distributions for the non-regression coefficient parameters. The choice of gamma distributions is based on their flexibility, as they allow for the inclusion of non-informative priors, such as the uniform distribution. For the regression coefficients, we set a prior scenario using a non-informative independent gamma distribution with parameters Gamma(10,10) as the baseline distribution. This choice is made because we lack any prior information from historical data or previous experiments. This selection is motivated by the fact that these priors have been widely considered in numerous study publications in the literature, including references [36–40].

$$\begin{array}{c} \pi \left(\lambda \right)∼G\left(a_{1},b_{1}\right)\text{,}\;\text{i.e.,}\;\text{with}\;\text{PDF}\;\;\frac{b_{1}^{a_{1}}}{\Gamma (a_{1})}\lambda^{a_{1}-1}e^{-b_{1}\lambda };\;a_{1},b_{1},\lambda >0 \end{array}$$ (68)

and

$$\begin{array}{c} \pi \left(\alpha \right)∼G\left(a_{2},b_{2}\right)\text{,}\;\text{i.e.,}\;\text{with}\;\text{PDF}\;\;\frac{b_{2}^{a_{2}}}{\Gamma (a_{2})}\alpha^{a_{2}-1}e^{-b_{2}\alpha };\;a_{2},b_{2},\alpha >0. \end{array}$$ (69)

The hyperparameter values of the prior distributions are selected based on historical data of the baseline distribution [36].

For the prior of regression coefficients, we have

$$\begin{array}{c} \pi \left(\beta^{\prime }\right)∼N\left(a_{3},b_{3}\right), \end{array}$$ (70)

where *β*′ = (*β*_1_, *β*_2_, …, *β*_*J*_) represents a vector of regression coefficients associated with the covariates in the HRF of the survival model.

The joint prior distribution for the baseline hazard parameters and regression coefficients is expressed as follows:

$$\begin{array}{c} \pi \left(\alpha ,\lambda ,\beta^{\prime }\right)=\pi \left(\alpha \right)\pi \left(\lambda \right)\pi \left(\beta^{\prime }\right). \end{array}$$ (71)

The model requires data *D* = (*t*_*i*_, *δ*_*i*_, *x*_*i*_), *i* = 1, …, *n*, where *t*_*i*_ represents the observed lifetime time for the *i*-th individual, *δ*_*i*_ denotes the censoring status (1 if the event of interest has occurred, 0 otherwise), and *x*_*i*_ represents the explanatory variables.

### 4.2 Posterior specification

Bayes’ theorem is employed to combine prior knowledge and experimental data in the posterior distribution as follows:

$$\begin{array}{c} P\left(h_{0},\beta ;D\right)\propto L\left(h_{0},\beta \right)\pi \left(h_{0},\beta \right), \end{array}$$ (72)

where *L*(*h*_0_, *β*) represents the likelihood function of (*h*_0_, *β*) as represented in Eq (51).

In this study, Bayesian inference is performed using the Stan software, which employs Hamiltonian Monte Carlo (HMC) algorithms. The Stan framework utilizes the No-U-Turn Sampler (NUTS) algorithm, an advanced variant of HMC, to efficiently sample from the posterior distribution (Hoffman and Gelman, 2014). The NUTS algorithm automatically tunes its parameters, such as the step size and trajectory length, during the sampling process to improve sampling efficiency [37, 41].

To update each parameter component, Stan employs a combination of HMC and the Gibbs sampling algorithm. This allows for efficient exploration of the posterior distribution while maintaining the conditional independence structure of the model. By incorporating the Gibbs sampling algorithm within the Stan framework, we can efficiently sample from the joint posterior distribution of the parameters [2].

### 4.3 McMC simulation

Calculating the marginal posterior distribution is difficult due to the complex high-dimensional integral involved. It is impossible to obtain accurate marginal distributions and the normalized joint posterior distribution analytically. Therefore, we employ the McMC simulation method, specifically utilizing the HMC (Hamiltonian Monte Carlo) method and its adaptive NUTS (No-U-Turn sampler) algorithm, to approximate these integrals. We present the implementation and configuration details of these methods. With the assistance of STAN, we use the McMC simulation method to carry out the estimation procedure and uncover significant discoveries [42, 43].

#### 4.3.1 HMC algorithm

The Hamiltonian Monte Carlo (HMC) algorithm is a powerful method for efficiently exploring the posterior distribution by utilizing derivatives of the target density function. It employs a simulation of Hamiltonian dynamics through numerical integration, followed by a Metropolis acceptance step, to draw samples from the joint density function involving both the parameter *θ* and auxiliary momentum variables *φ*. In this sub-section, we present the HMC technique using the notation introduced by [44], aligning it with [43, 45, 46], with the goal of sampling from a density function denoted as *p*(*θ*), representing the parameter *θ* in Bayesian analysis, often expressed as a Stan program for the Bayesian posterior *p*(*θ*|*x*) conditioned on observed data *X*.

To begin, a multivariate normal distribution is commonly used as the auxiliary density, with *φ* being drawn from *φ* ∼ MultiNormal(0, *ϵ*). It is important to note that this auxiliary density is independent of the *θ* parameters. The parameter *ϵ* represents the Euclidean metric and acts as a measure of variability. By transforming the parameter space, this auxiliary density enables more effective and efficient sampling. In Stan, the inverse of *ϵ* (*ϵ*^−1^) is typically set to a diagonal estimate of the covariance computed during the warm-up phase.

The joint density function *p*(*φ*, *θ*) defines the Hamiltonian, denoted as *Q*(*φ*, *θ*) = −log(*p*(*φ*, *θ*)) or equivalently *Q*(*φ*, *θ*) = −log(*p*(*φ*|*θ*)) − log(*p*(*θ*)), which can also be expressed as *Q*(*φ*, *θ*) = *K*(*φ*|*θ*) + *P*(*θ*). Here, *K*(*φ*|*θ*) represents the kinetic energy, and *P*(*θ*) represents the potential energy. In a Stan program, the log density is defined to describe the characteristics of the distribution based on the current parameter value *θ*.

The transition to a new state occurs in two stages before undergoing a Metropolis acceptance step. Firstly, the momentum *φ* is independently sampled as *φ* ∼ MultiNormal(0, *ϵ*) and is not carried over between iterations. Next, Hamilton’s equations are utilized to evolve the joint system, which includes both the current parameter values *θ* and the newly sampled momentum *φ*. The equations governing this evolution are given by:

$$\begin{array}{c} \frac{d\theta }{dt}=\frac{dQ}{d\varphi },\;\frac{d\varphi }{dt}=-\frac{dQ}{d\theta }. \end{array}$$ (73)

Since the momentum density is independent of the target density (i.e., *p*(*φ*|*θ*) = *p*(*φ*)), the first term in the time derivative of the momentum, $\frac{dK(\varphi |\theta )}{d\theta }$, becomes zero, resulting in zero contributions to the pair of time derivatives.

Following previous implementations of Hamiltonian Monte Carlo (HMC), Stan employs the leapfrog integrator to solve this two-state differential equation. The leapfrog algorithm, a numerical technique designed for stable results in Hamiltonian systems, operates by taking discrete steps with a small time interval denoted as *ϵ*. The algorithm starts by independently sampling a new momentum term, *φ* ∼ MultiNormal(0, *ϵ*), without relying on the parameter values *θ* or the previous momentum value. Subsequently, the algorithm alternates between half-step updates of the momentum and full-step updates of the position:

$$\begin{array}{c} \varphi \leftarrow \varphi -\frac{\epsilon }{2}\frac{dP(\theta )}{d\theta },\;\theta \leftarrow \theta +\epsilon \epsilon^{-1}\varphi . \end{array}$$ (74)

By accumulating a simulated time of *Lϵ* through *L* leapfrog steps, a final state (*φ*,*θ*^)^ is obtained. If the leapfrog integrator were perfect in terms of numerical accuracy, introducing randomness solely through generating a random momentum vector for each transition would be sufficient. However, to account for numerical integration errors, a Metropolis acceptance step is included. This step determines the probability of accepting the proposal (*φ*, *θ*) generated from transitioning from the current state (*φ*,*θ*^)^. The acceptance probability is calculated as:

$$\begin{array}{c} s=\text{min}(1,\text{exp}(Q\left(\varphi ,\theta \right)-Q\left(\varphi^{,}\theta^{)}\right)). \end{array}$$ (75)

If the proposal is rejected, theprevious parameter value is saved and utilized to initiate the following iteration.

In summary, the HMC algorithm begins by initializing a preset parameter set *θ*, either provided by the user or randomly generated in the Stan framework. After a set number of iterations, a new momentum vector is sampled, and the leapfrog integrator updates the current parameter value *θ*. Leapfrog integration, based on Hamiltonian dynamics, is performed with a discretization time interval *ϵ* and a specified number of steps (*L*). Following this, an acceptance step, guided by the Metropolis criterion, determines whether to transition to the new state (*φ*,*θ*^)^ or maintain the current state.

### 4.4 Model specification

Suppose that observed data *y*_1_, …, *y*_*n*_, are modeled as independent given parameters Ψ. This implies that $P\left(Y|\Psi \right)=\prod_{i=1}^{n}P\left(y_{i}|\Psi \right)$. Then assume we have a prior distribution *P*(Ψ), which results in a posterior distribution *P*(Ψ|*Y*) and a posterior predictive distribution $P(\tilde{Y})$. By employing Bayes’ theorem, we can express the joint or pointwise posterior density as follows:

$$\begin{array}{c} P(\Psi |Y)\propto L(\Psi |Y)\times P(\Psi ), \end{array}$$ (76)

where the posterior is proportional to the likelihood multiplied by the prior, where Ψ represents the model parameters and *Y* denotes the observed data.

### 4.5 The expected log predictive density

Consider data *y*_1_, *y*_2_, .., *y*_*n*_, which is independent given parameters Ψ. Thus the likelihood can be decomposed into the following product of pointwise likelihoods:

$$\begin{array}{c} P\left(y|\Psi \right)=\prod_{i=1}^{n}P\left(y_{i}|\Psi \right). \end{array}$$

Suppose a prior distribution *P*(Ψ) and posterior predictive distribution for new data $\tilde{y_{i}}$, then we have

$$\begin{array}{c} P\left(\tilde{y}|y\right)=\int P\left(\tilde{y_{i}}|\Psi \right)P\left(\Psi |y\right)d\Psi . \end{array}$$

The expected log predictive density (ELPD) is given by

$$\begin{array}{c} elpd=\int \text{log}\;P\left(\tilde{y_{i}}|y_{i}\right)P\left(\tilde{y_{i}}\right)d\tilde{y_{i}}, \end{array}$$ (77)

where $\text{log}\;P(\tilde{y_{i}}|y_{i})$ is the log predictive density of the model for a new observation $\tilde{y_{i}}$, that has been generated by some true, unknown process, $P(\tilde{y_{i}})$. The ELPD is a measure of out-of-sample predictive performance for a model. It is defined as the average of the log predictive densities of all possible future data points, where the log predictive density is the log-likelihood of a data point under the model. The ELPD can be estimated using Bayesian methods such as LOOIC or WAIC, which estimate the predictive density for each data point using the posterior distribution of the model parameters and then averaging over the posterior distribution of the data-generating process.

### 4.6 Out-of-sample pointwise predictive accuracy estimation

For a new data point $\tilde{y_{i}}$, the out-of-sample predictive fit is indicated as follows:

$$\begin{array}{c} \text{log}\;P_{post}\left(\tilde{y_{i}}\right)=\text{log}\;E_{post}\left(\tilde{y_{i}}\right)=\text{log}\int P\left(\Psi |\tilde{y_{i}}\right)P_{post}\left(\Psi \right)d\Psi , \end{array}$$ (78)

where $P_{post}\left(\tilde{y_{i}}\right)$ represents the predictive density for $\tilde{y_{i}}$, which is generated from the posterior distribution *P*_*post*_(Ψ). Note that, we use the notation *P*_*post*_ to indicate the posterior distribution.

### 4.7 The Watanabe-Akaike Information Criteria (WAIC)

The WAIC is a Bayesian information criteria used to evaluate statistical models’ out-of-sample predictive accuracy [11, 13]. It is less sensitive to model overfitting and employs a more fully Bayesian approach than other information criteria, such as the Akaike information criterion (AIC). To construct the WAIC, we first compute the log pointwise posterior predictive density for each data point *d*_*i*_. This is accomplished by averaging the log predictive density over the model parameters’ posterior estimates. The variance of the log predictive density is then calculated for every data point. In order to determine the effective number of parameters, we sum the variance across all the data points.

A model’s complexity is measured by its effective number of parameters. It refers to the number of model parameters that are utilized to accurately predict the data. It is less likely for a model to overfit the data if it has fewer effective parameters. After determining the effective number of parameters, we can compute WAIC using the following formula:

$$\begin{array}{c} P_{WAIC}=2\sum_{i=1}^{n}(\text{log}\left(E_{post}P\left(y_{i}|\Psi \right)\right)-E_{post}\left(\text{log}\;P\left(y_{i}|\Psi \right)\right)), \end{array}$$ (79)

where the expectation *E*_*post*_ is an average of Ψ over its posterior distribution. The *elpd*_*WAIC*_ is thus obtained as the following difference:

$$\begin{array}{c} elpd_{WAIC}=elpd-P_{WAIC}. \end{array}$$ (80)

After that, we get the WAIC defined as

$$\begin{array}{c} WAIC=-2elpd_{WAIC}. \end{array}$$ (81)

The WAIC can be used to assess different models and determine which model is most likely to accurately predict the data. The optimal model is one with a lower WAIC value.

### 4.8 The Leave-One-Out Information Criteria (LOOIC)

The LOOIC is a model selection criterion that estimates the out-of-sample predictive performance of a statistical model. It is based on the idea of leaving out one observation at a time, fitting the model to the remaining data, and then using the fitted model to predict the left-out observation. This process is repeated for all observations in the dataset, and the average of the predicted log-likelihoods is used to assess the model’s out-of-sample predictive performance. The model with the lowest LOOIC is considered to be the best model.

The leave-one-out (LOO) technique is a method used to estimate the ELPD or generalization performance of a model. It involves training the model on all observations except for a particular observation *y*_*i*_, and then predicting the held-out observation *y*_*i*_. This process is repeated for each of the *n* observations, treating each observation *y*_*i*_ as a pseudo-Monte Carlo sample from the true generating model *p*_*t*_. Consequently, we obtain *n* LOO posterior distributions, denoted as *p*(Ψ|*y*_−*i*_), where *y*_−*i*_ represents the data with observation *y*_*i*_ removed [12].

Using the LOO posteriors, we can estimate the ELPD, as shown in Eq (77), using the following formula:

$$\begin{array}{c} elpd_{loo}=\frac{1}{n}\sum_{i=1}^{n}\;\text{log}\;P\left(y_{i}|y_{-i}\right) \end{array}$$ (82)

$$\begin{array}{c} =\frac{1}{n}\sum_{i=1}^{n}\;\text{log}\int P\left(y_{i}|\Psi \right)P\left(\Psi |y_{-i}\right)d\Psi , \end{array}$$ (83)

where *P*(*y*_*i*_|Ψ) represents the likelihood, and *P*(Ψ|*y*_−*i*_) denotes the posterior distribution for Ψ when we exclude the observation *y*_*i*_.

## 5 Practical applications

**Fig 4:**
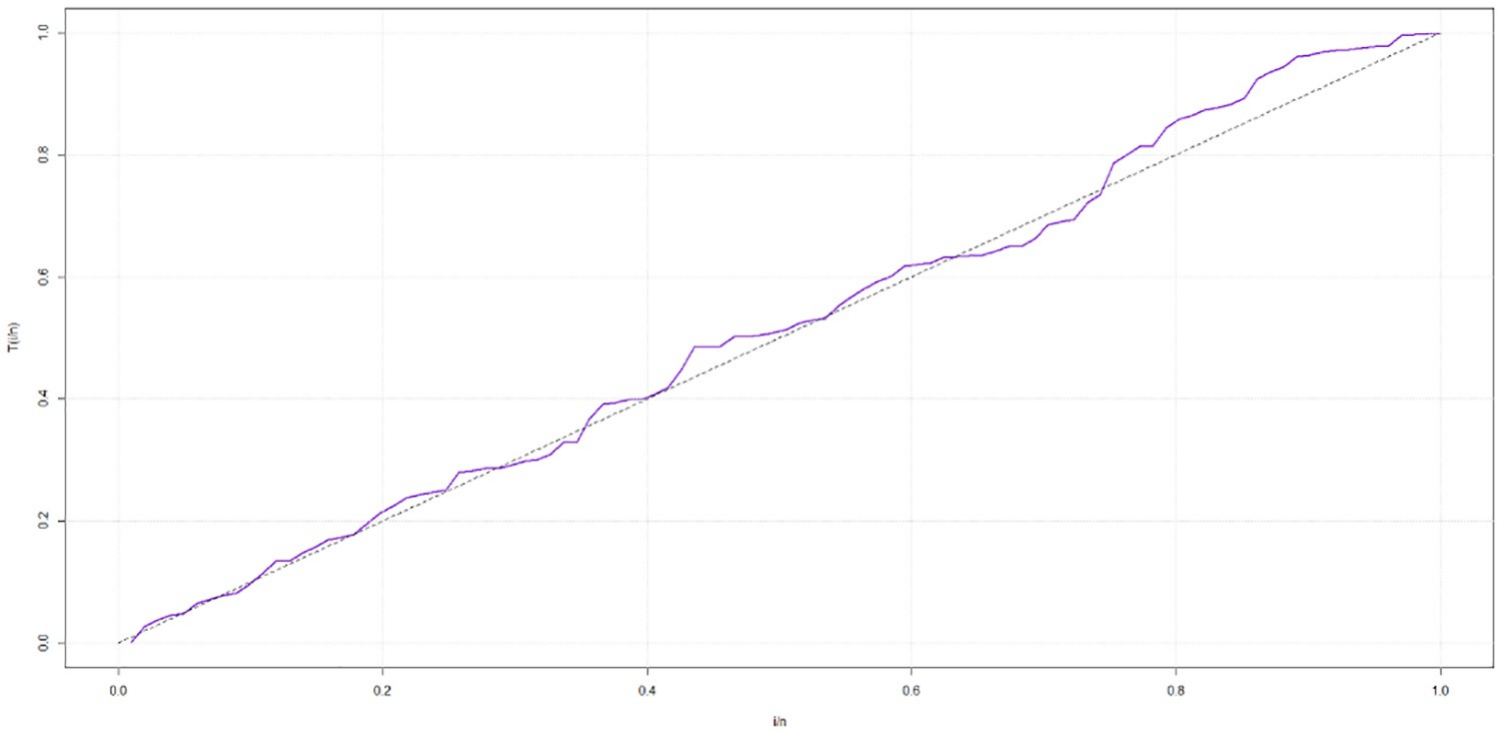
TTT plot for the gastric cancer data set.

**Fig 5:**
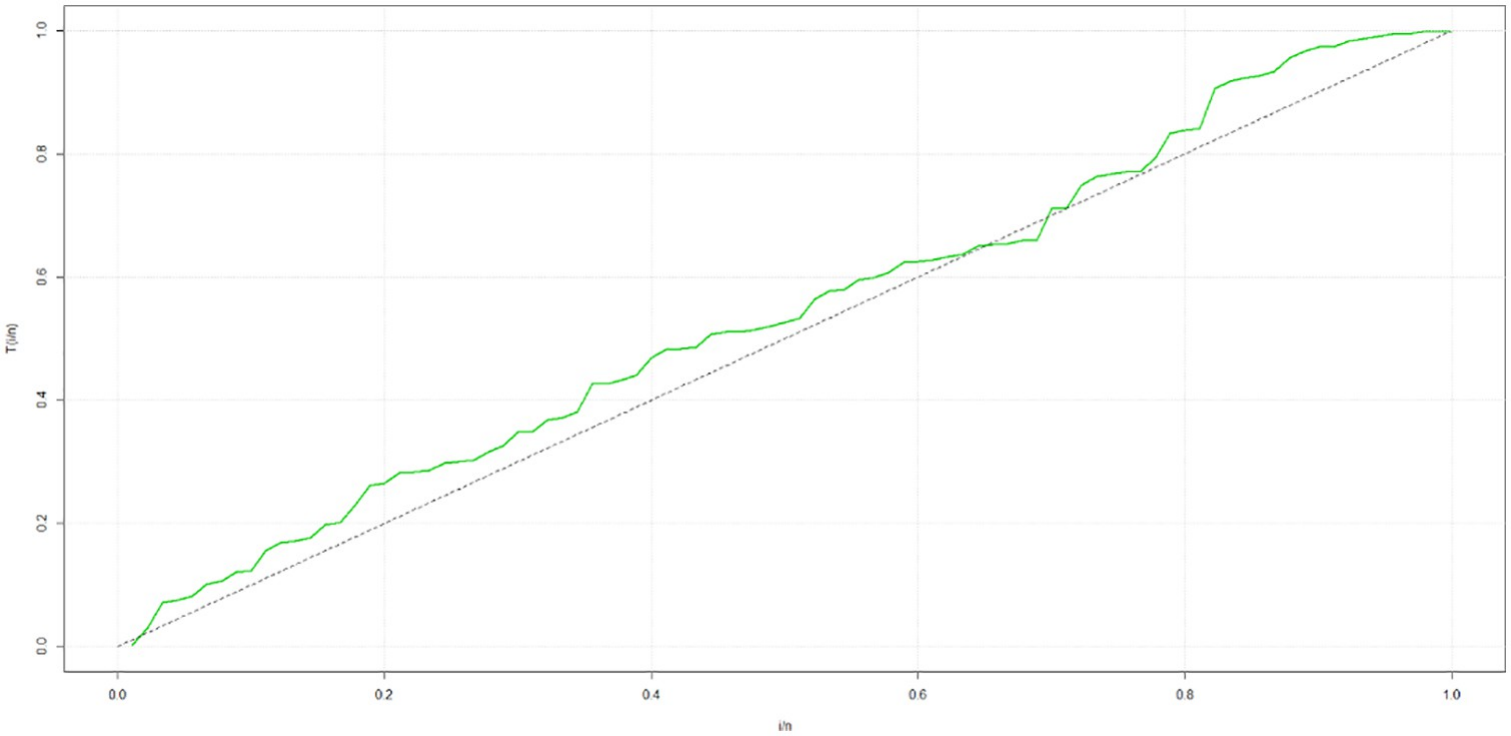
TTT plot for the Alloauto data set.

This research utilizes two datasets sourced from existing literature to showcase the effectiveness of the sin-G family of baseline distributions for modeling censored survival data. These two datasets exhibit contrasting hazard rate function (HRF) shapes, with one dataset displaying a monotonic HRF shape as shown in Fig 4 and the other featuring a non-monotonic HRF shape as shown in Fig 5.

### 5.1 Data I

In this section, we analyze a dataset from a clinical trial in gastric oncology that contains right-censored data. Our objective is to demonstrate the application of the proposed parametric PH model to modeling lifetime data with right-censored datasets. Initially, we employ Bayesian analysis using the Rstan package to examine the PH model and its competing baseline hazards, such as the SW, SG, SL, and SEE distributions. Subsequently, we compare the models using two evaluation metrics: the WAIC and the LOOIC. Finally, we interpret the obtained results.

#### 5.1.1 Data source and description

In this analysis, we examine the gastric cancer data collected from the Gastrointestinal Tumor Study Group (1982). This dataset has been widely considered in studies focusing on crossing survival curves, particularly in the field of survival analysis. Some notable studies that have utilized this dataset include Demarqui and Mayrink [47], Muse et al. [17], and Diao et al. [48]. The dataset, labeled “gastric,” is freely accessible through the R package AmoudSurv [49].

The clinical trial in oncology comprises 90 patients diagnosed with locally advanced gastric cancer. These patients were randomly divided into two groups: (i) a control group consisting of 45 patients who received chemotherapy, and (ii) a treatment group consisting of 45 patients who underwent radiation therapy in addition to chemotherapy. The study followed these patients for a duration of approximately 5 years. Each patient’s data in the dataset includes three variables: the response time, which indicates either failure (time to death) or right censoring; a binary failure indicator identifying patients who experienced the event of interest; and a binary group indicator (1) indicating the type of treatment received.

#### 5.1.2 Bayesian analysis application

In this part, we employ the McMC samples of posterior properties for the proposed fully parametric PH models with the Sine-G baseline distributions.

#### 5.1.3 Results

Table 1 presents the results, allowing us to assess various posterior characteristics of interest and their corresponding numerical values.

**Table 1:** Results for the posterior properties of the SW-PH, SL-PH, SG-PH and SEE-PH models.

Models	Par (s)	Estimate	SE	SD	2.5%	Medium	97.5%	*N*_*eff*_	$\hat{R}$
SW-PH	*β*	-0.056	0.004	0.221	-0.491	-0.058	0.383	3739	1.000
	λ	0.340	0.001	0.048	0.252	0.338	0.441	3576	1.001
	*α*	0.951	0.001	0.083	0.797	0.949	1.119	4138	1.000
SL-PH	*β*	0.079	0.004	0.214	-0.342	0.078	0.494	3237	1.000
	λ	0.589	0.003	0.157	0.341	0.571	0.958	2866	1.000
	*α*	0.872	0.003	0.175	0.578	0.855	1.265	2705	1.001
SG-PH	*β*	0.080	0.004	0.223	-0.353	0.080	0.513	2992	1.000
	λ	0.208	0.001	0.037	0.143	0.206	0.289	2567	1.001
	*α*	1.077	0.005	0.260	0.644	1.053	1.661	2510	1.000
SEE-PH	*β*	-0.050	0.004	0.214	-0.479	-0.047	0.373	3498	1.001
	λ	0.353	0.001	0.058	0.247	0.351	0.472	2766	1.000
	*α*	1.040	0.002	0.119	0.821	1.035	1.295	2684	1.001

Table 1 summarizes the posterior properties of four different models: SW-PH, SL-PH, SG-PH, and SEE-PH. It provides estimates, standard errors, quantiles, effective sample sizes (*N*_*eff*_), and potential scale reduction factors ($\hat{R}$) for each model’s parameters. The results indicate the estimated values and uncertainties of the parameters, while *N*_*eff*_ and $\hat{R}$ provide information about the convergence of the Markov chain Monte Carlo algorithm. These findings are essential for understanding the posterior distributions and making inferences about the models’ parameters.

#### 5.1.4 Convergence diagnostics

To assess the convergence of the algorithm for the proposed regression models, we employed numerical and visual methods. The HMC-NUTS algorithm, utilized in the McMC procedure, has successfully converged to the joint posterior distribution, as demonstrated by the summary results provided in the table above. Several essential indicators support this conclusion: the potential scale reduction factor $\hat{R}$ is 1, the effective sample size (*n*_*eff*_) exceeds 400, and the Monte Carlo error (SE) is less than 0.05 of the posterior standard deviations for all parameters.

**Fig 6:**
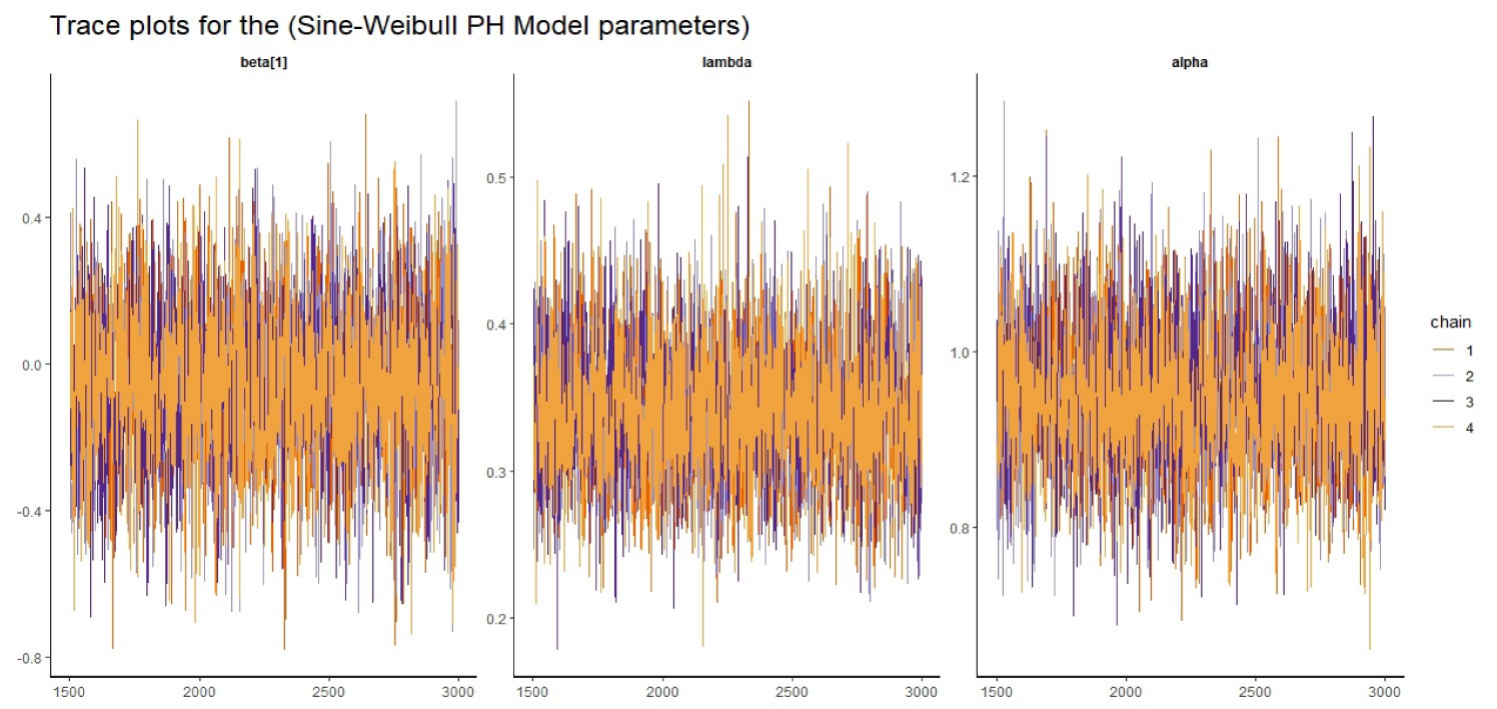
The trace plots of the posterior parameters for the SW-PH model using gastric cancer data.

**Fig 7:**
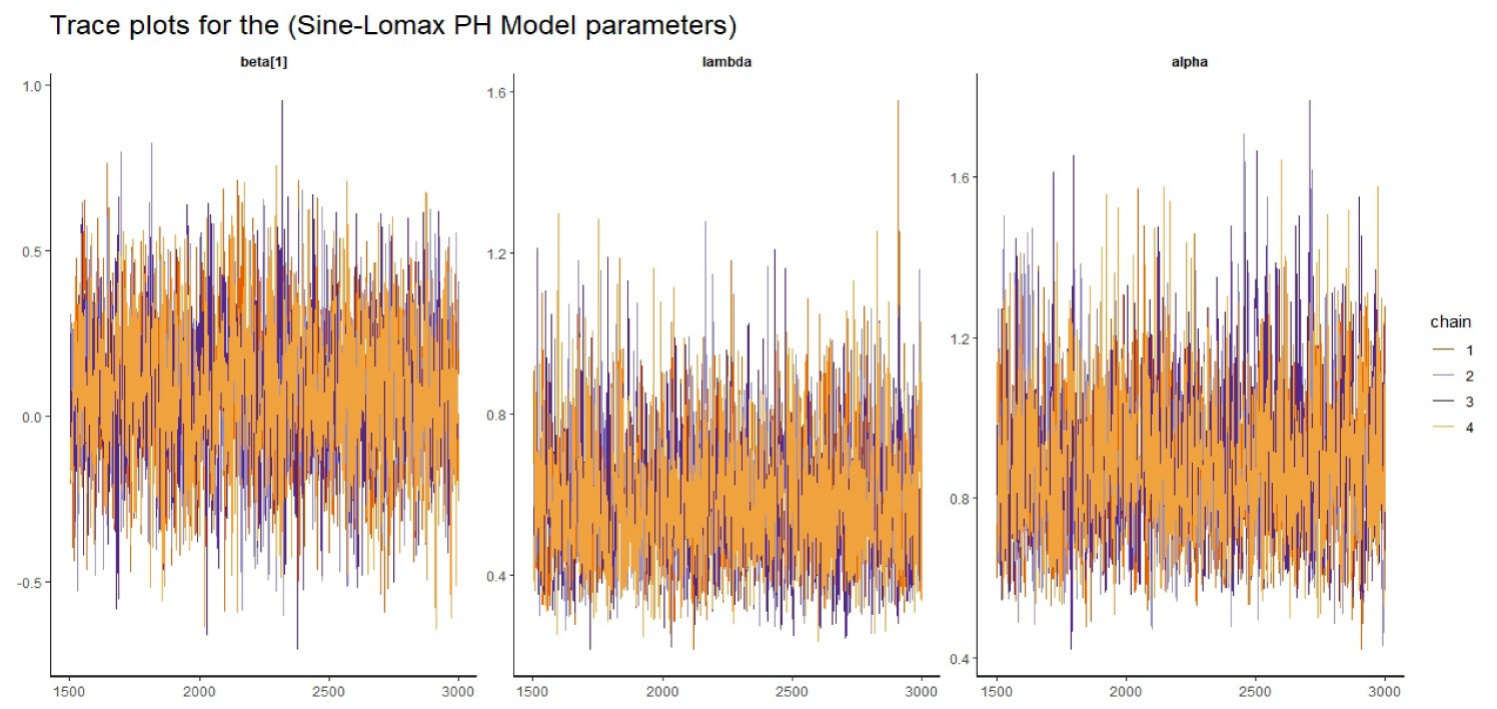
The trace plots of the posterior parameters for the SL-PH model using gastric cancer data.

**Fig 8:**
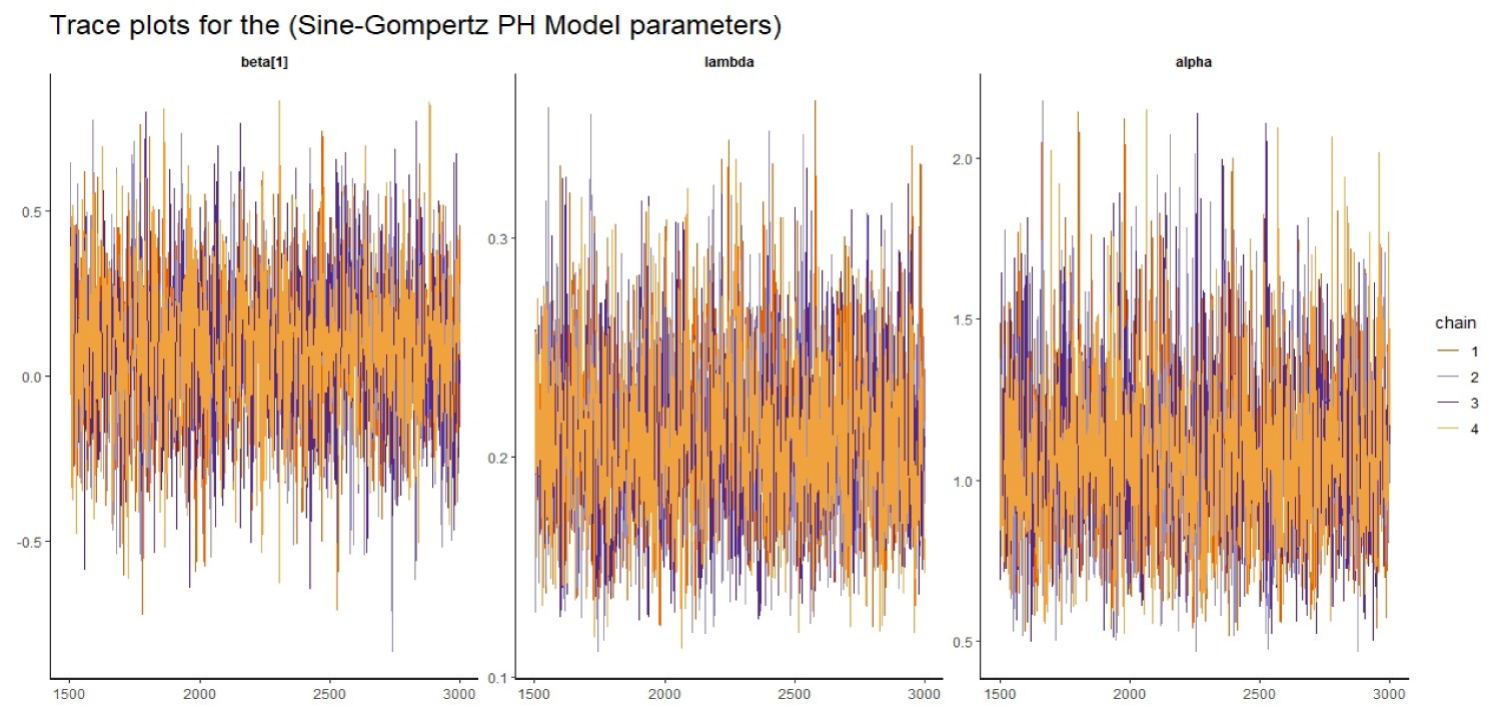
The trace plots of the posterior parameters for the SG-PH model using gastric cancer data.

**Fig 9:**
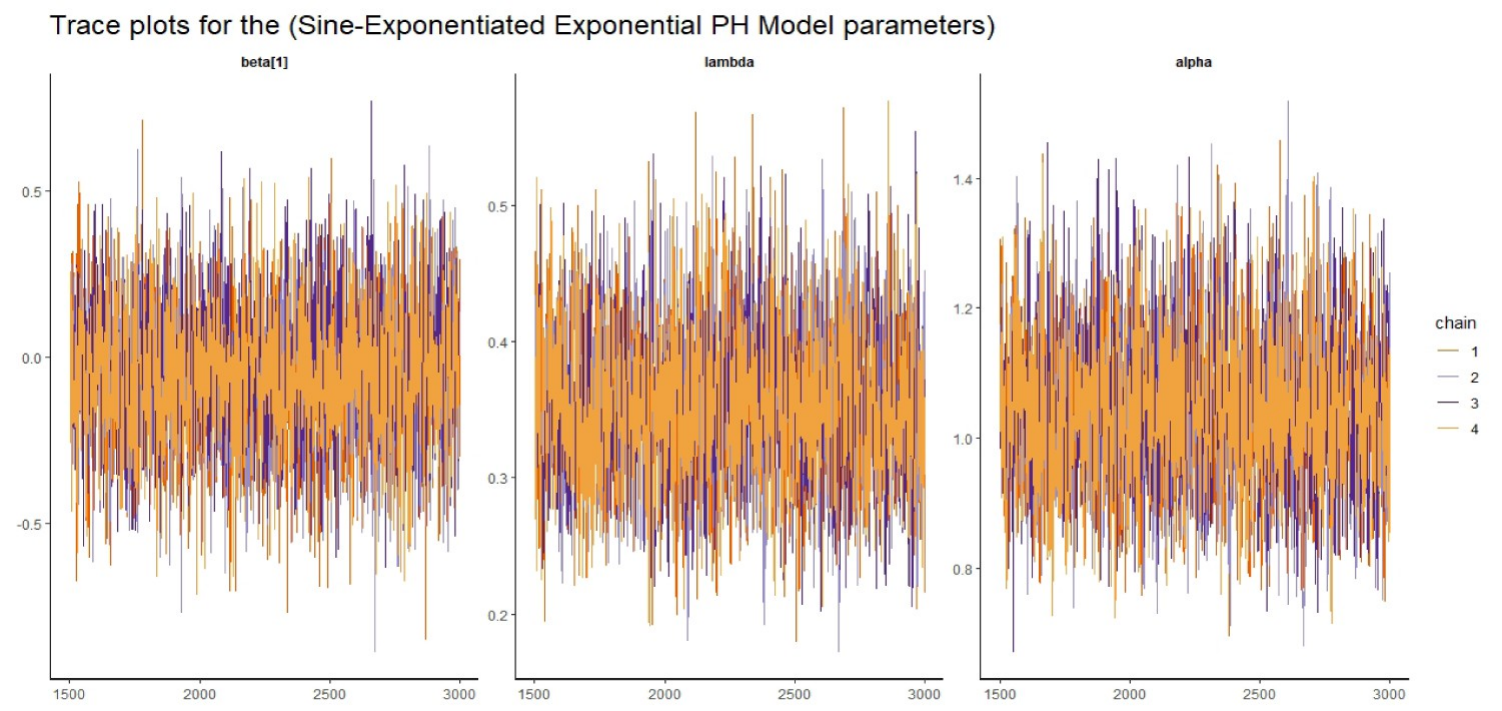
The trace plots of the posterior parameters for the SEE-PH model using gastric cancer data.

**Fig 10:**
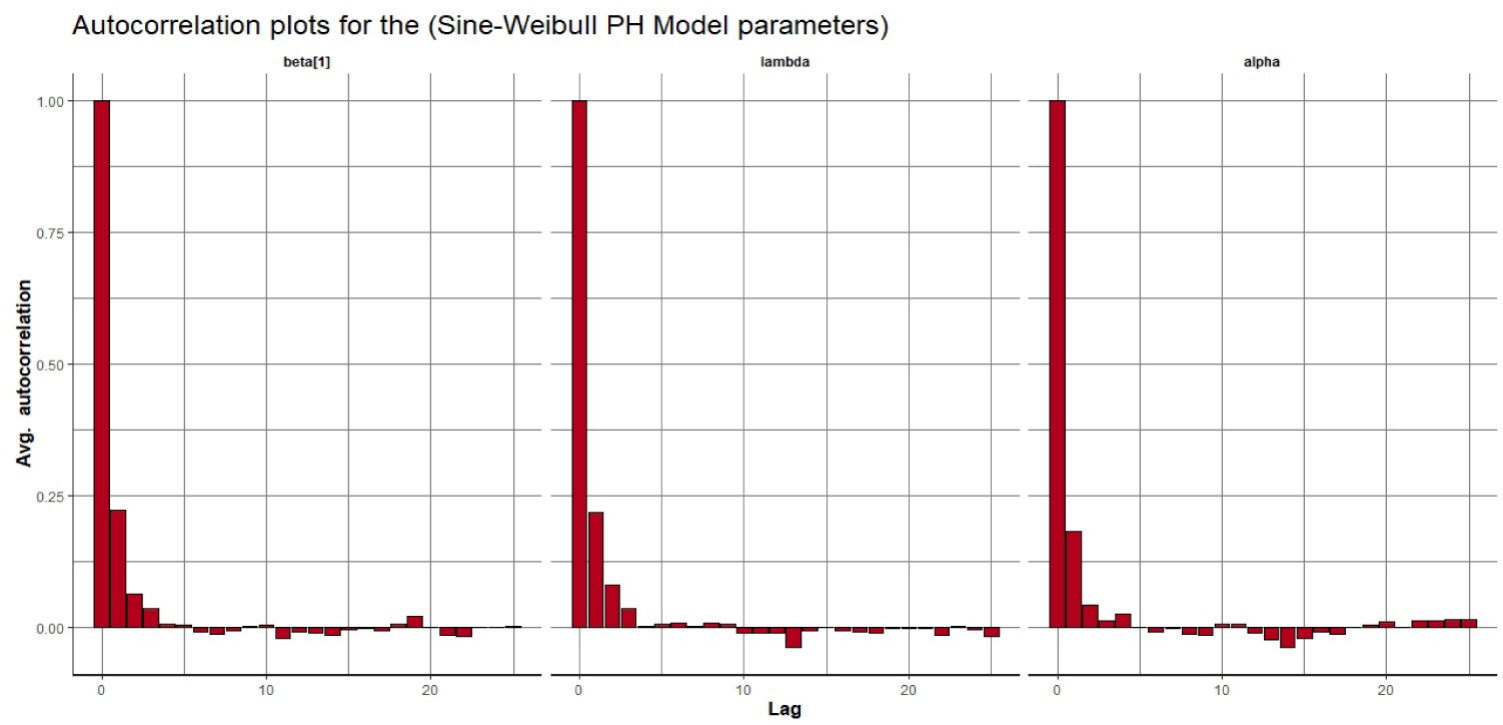
The autocorrelation plots of the posterior parameters for the SW-PH model using gastric cancer data.

**Fig 11:**
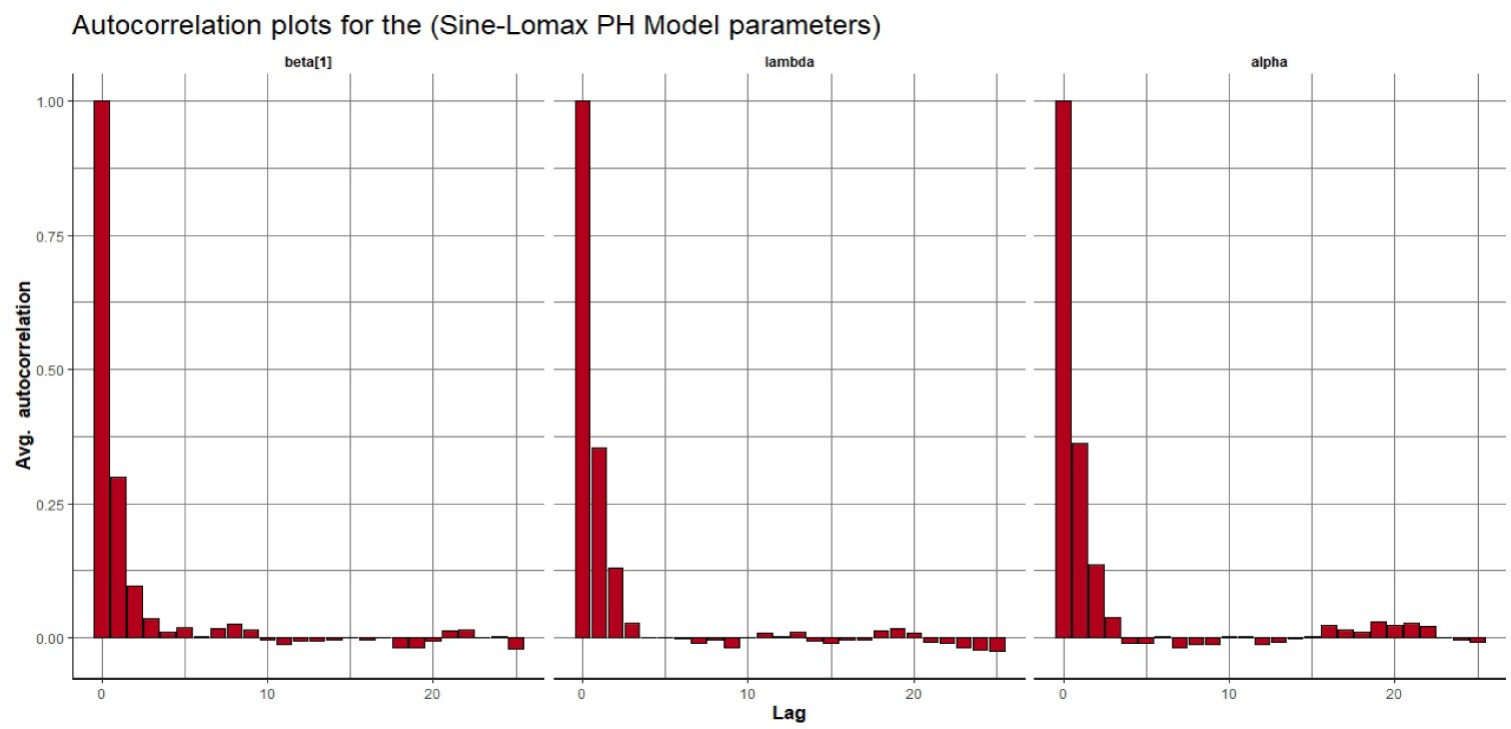
The autocorrelation plots of the posterior parameters for the SL-PH model using gastric cancer data.

**Fig 12:**
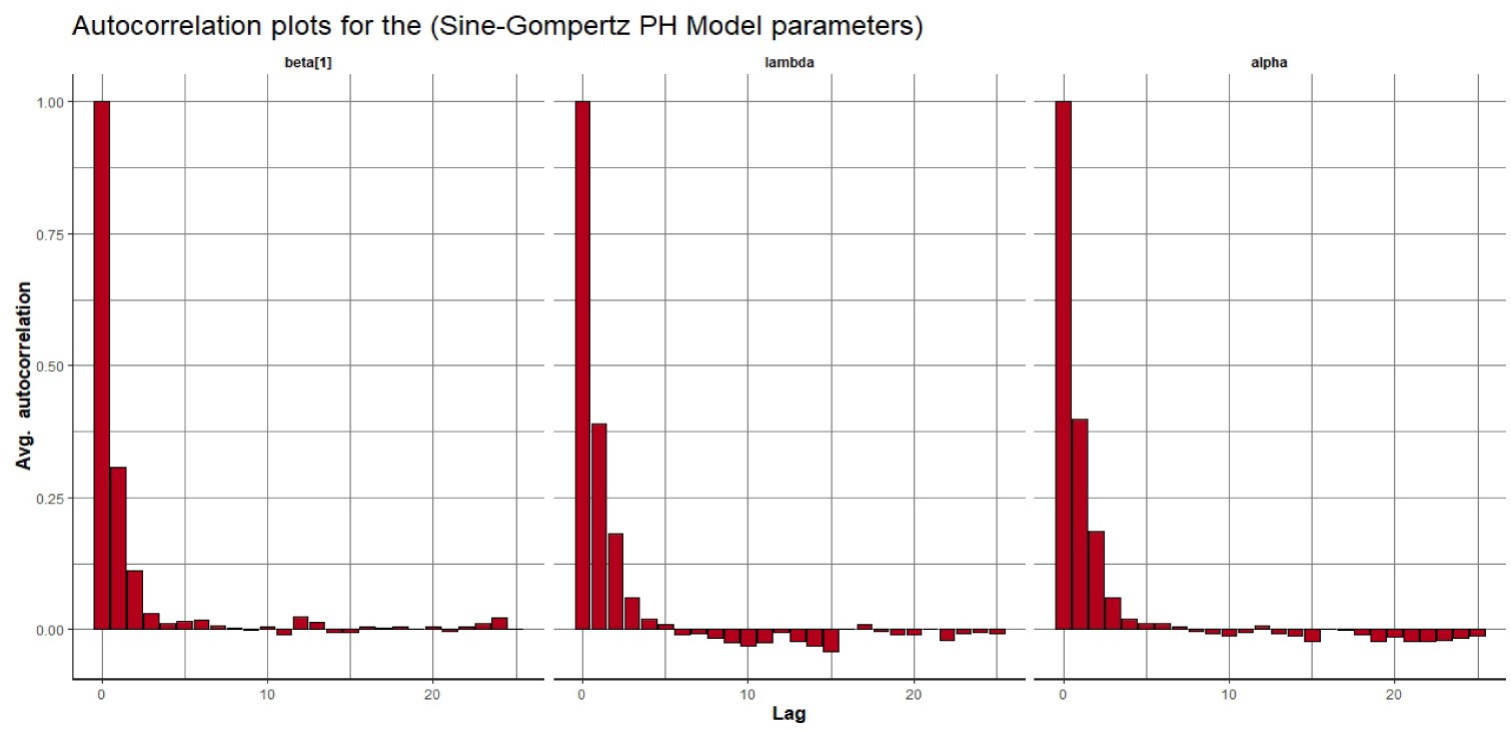
The autocorrelation plots of the posterior parameters for the SG-PH model using gastric cancer data.

**Fig 13:**
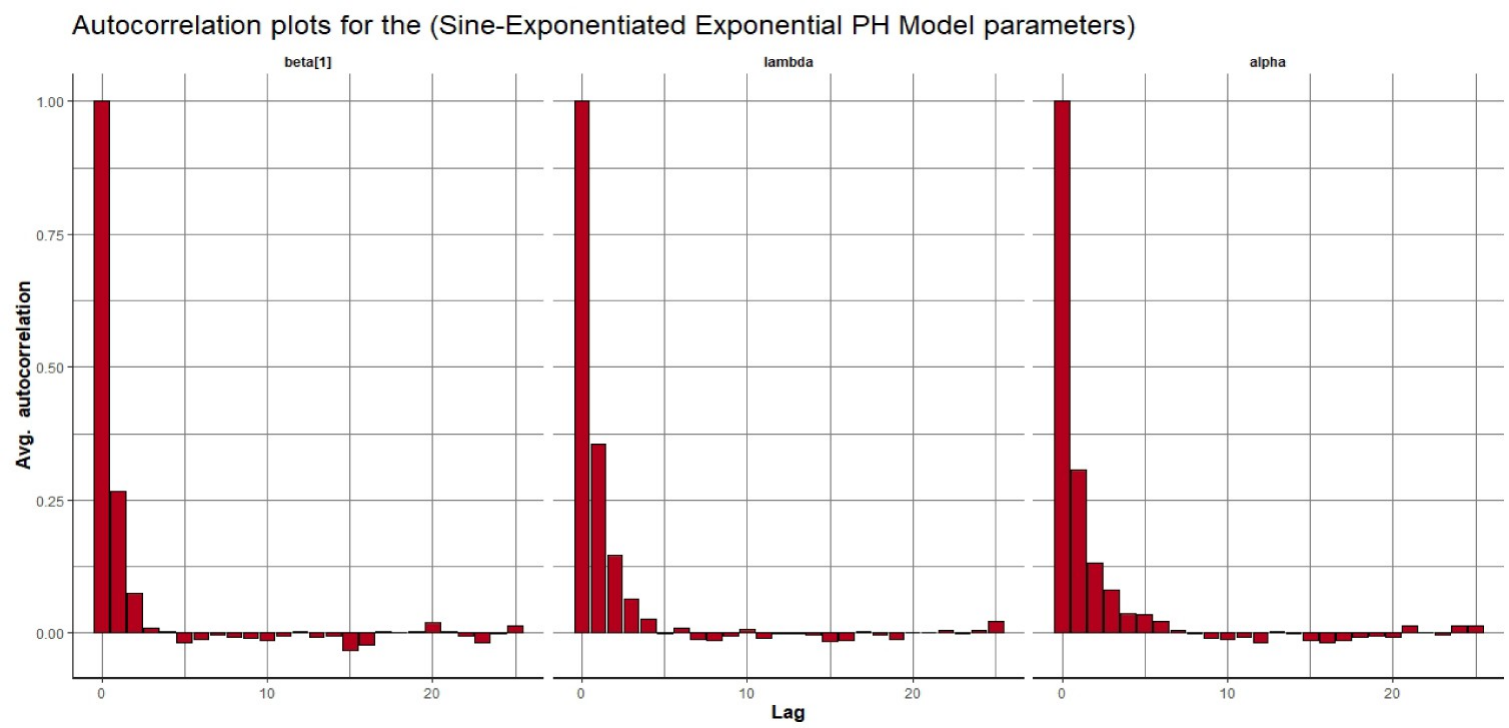
The autocorrelation plots of the posterior parameters for the SEE-PH model using gastric cancer data.

Visual assessment of convergence is commonly performed by examining kernel density, auto-correlation, and trace graphs [17, 45]. Figs 6–9 illustrate a stationary pattern fluctuating within a defined range, providing visual evidence of the McMC algorithm’s convergence. Additionally, Figs 10–13 displaying the auto-correlation plots, demonstrate a rapid decrease in auto-correlation as the lag period increases. This indicates satisfactory mixing and convergence of the algorithm towards the desired posterior distribution.

Table 2 presents the Bayesian model comparison results for four different models: SW-PH, SL-PH, SG-PH, and SEE-PH models. The comparison is based on two evaluation metrics: the WAIC and the LOOIC. The WAIC and LOOIC values provide a measure of the models’ goodness of fit and complexity. Lower values indicate better model performance.

**Table 2:** Bayesian model comparison for the SW-PH, SL-PH, SG-PH, and SEE-PH models.

Model	WAIC	LOOIC
SW-PH	258.30	258.35
SL-PH	256.40	256.40
SG-PH	269.20	269.20
SEE-PH	258.90	258.80

From the Table 3, we can observe the following:

- The SL-PH model has the lowest WAIC and LOOIC values, indicating the best overall fit to the data among the four models.
- The SW-PH model also shows relatively low WAIC and LOOIC values, suggesting a good fit to the data.
- The SEE-PH model and SG-PH model have higher WAIC and LOOIC values compared to the other models, indicating poorer fit or higher complexity.

**Table 3:** Results for the posterior properties of the SW-PH, SL-PH, SG-PH and SEE-PH models using dataset II.

Models	Par (s)	Estimate	SE	SD	2.5%	Medium	97.5%	*N*_*eff*_	$\hat{R}$
SW-PH	*β*	0.089	0.006	0.295	-0.493	0.094	0.667	2077	1.002
	λ	0.010	0.000	0.008	0.002	0.008	0.030	2058	1.002
	*α*	0.655	0.002	0.082	0.501	0.652	0.825	2418	0.999
SL-PH	*β*	-0.507	0.005	0.234	-0.961	-0.514	-0.037	2356	1.000
	λ	0.455	0.003	0.161	0.210	0.432	0.838	2758	1.002
	*α*	0.376	0.003	0.125	0.182	0.358	0.670	2189	1.000
SG-PH	*β*	0.230	0.004	0.223	-0.353	0.080	0.043	2349	1.000
	λ	0.454	0.001	0.027	0.152	0.316	0.209	2447	1.001
	*α*	1.031	0.005	0.260	0.644	1.053	1.541	23410	1.000
SEE-PH	*β*	-0.770	0.006	0.269	-1.301	-0.770	-0.242	2054	1.002
	λ	0.044	0.000	0.018	0.018	0.041	0.087	1952	1.004
	*α*	0.729	0.002	0.109	0.5311	0.722	0.966	2658	1.000

Based on these results in Table 2, the SL-PH model appears to be the most suitable choice for modeling the data, followed closely by the SEE-PH model. However, further analysis and consideration of other factors related to the specific research context may be necessary to make a final model selection.

### 5.2 Data II

Klein and Moeschberger [50] conducted a research study that involved 101 patients diagnosed with advanced acute myelogenous leukemia. Out of these patients, 51 underwent an autologous (auto) bone marrow transplant, while 50 received an allogeneic (allo) transplant. For 28 patients who underwent auto transplant and 22 patients who had allo transplant, the survival times (measured in months) were censored. By closely examining the TTT plot depicted in Fig 5, there is a notable suggestion of the hazard function’s unimodal nature.

#### 5.2.1 Results

Table 3 presents the results, allowing us to assess various posterior characteristics of interest and their corresponding numerical values using dataset II.

Table 3 summarizes the posterior properties of four different models: SW-PH, SL-PH, SG-PH, and SEE-PH. It provides estimates, standard errors, quantiles, effective sample sizes (*N*_*eff*_), and potential scale reduction factors ($\hat{R}$) for each model’s parameters. The results indicate the estimated values and uncertainties of the parameters, while *N*_*eff*_ and $\hat{R}$ provide information about the convergence of the Markov chain Monte Carlo algorithm. These findings are essential for understanding the posterior distributions and making inferences about the models’ parameters.

#### 5.2.2 Convergence diagnostics

**Fig 14:**
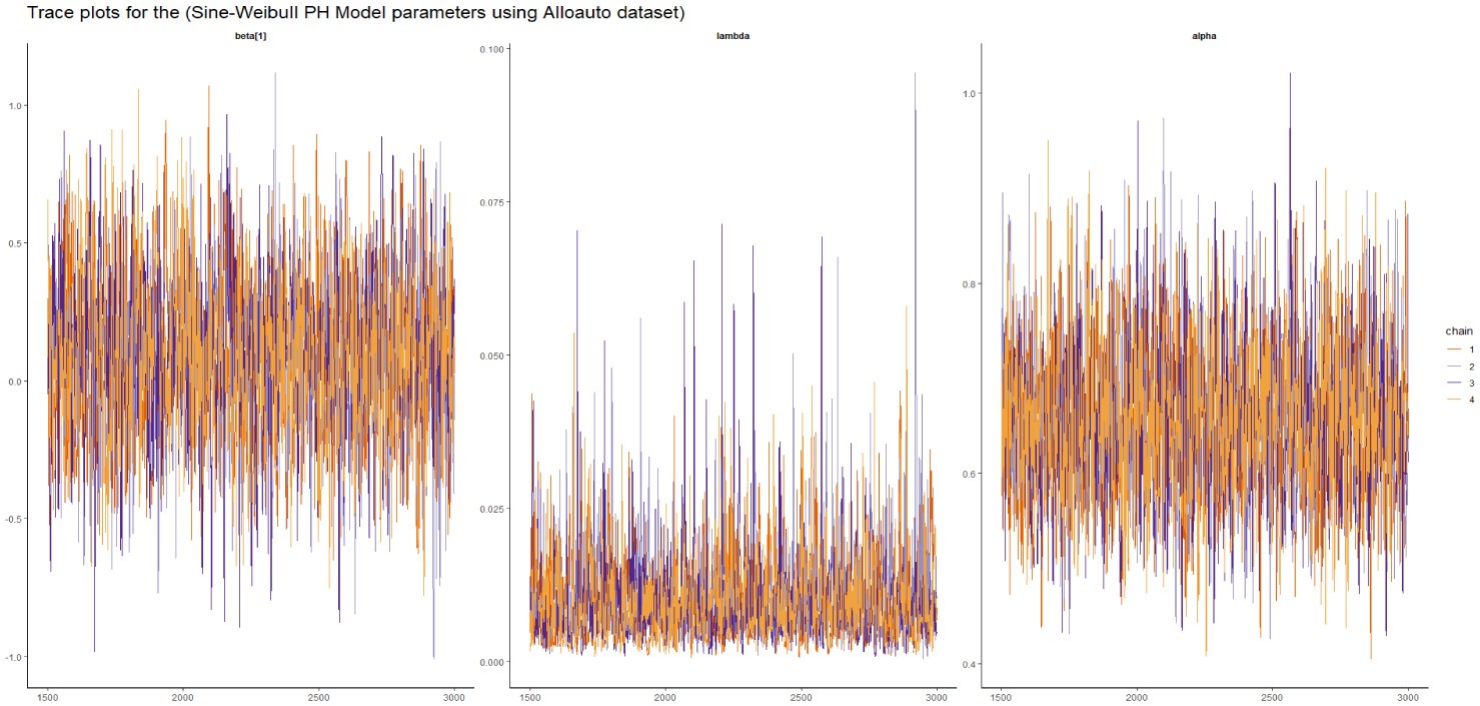
The trace plots of the posterior parameters for the SW-PH model using Alloauto data.

**Fig 15:**
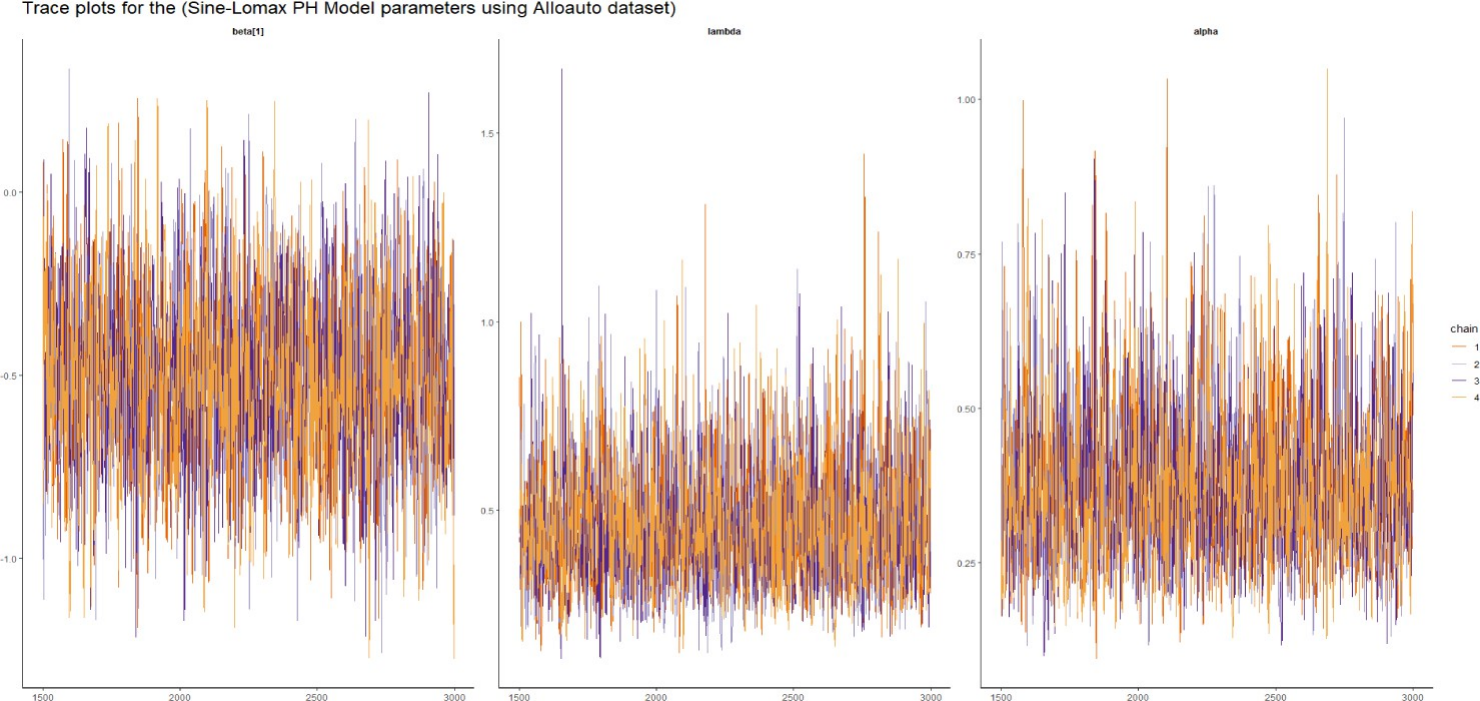
The trace plots of the posterior parameters for the SL-PH model using Alloauto data.

**Fig 16:**
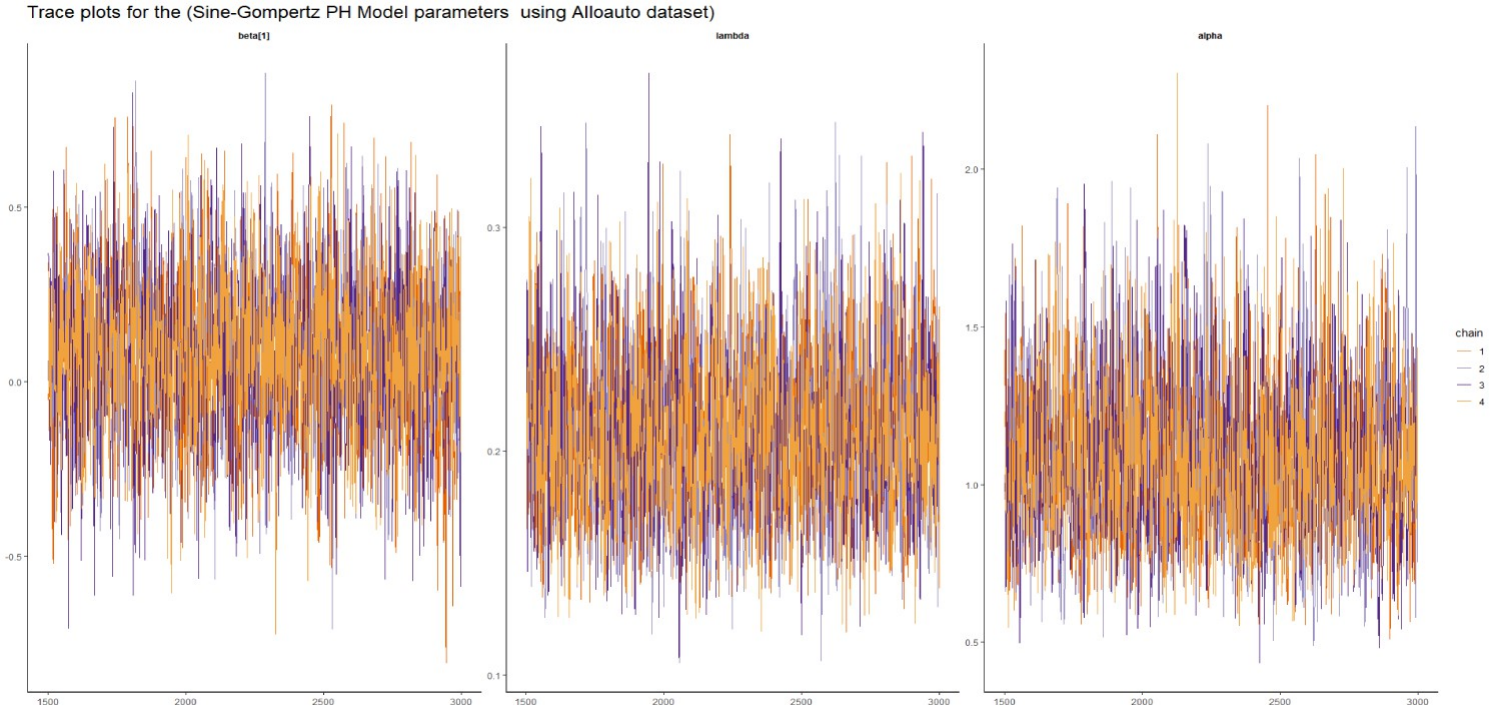
The trace plots of the posterior parameters for the SG-PH model using Alloauto data.

**Fig 17:**
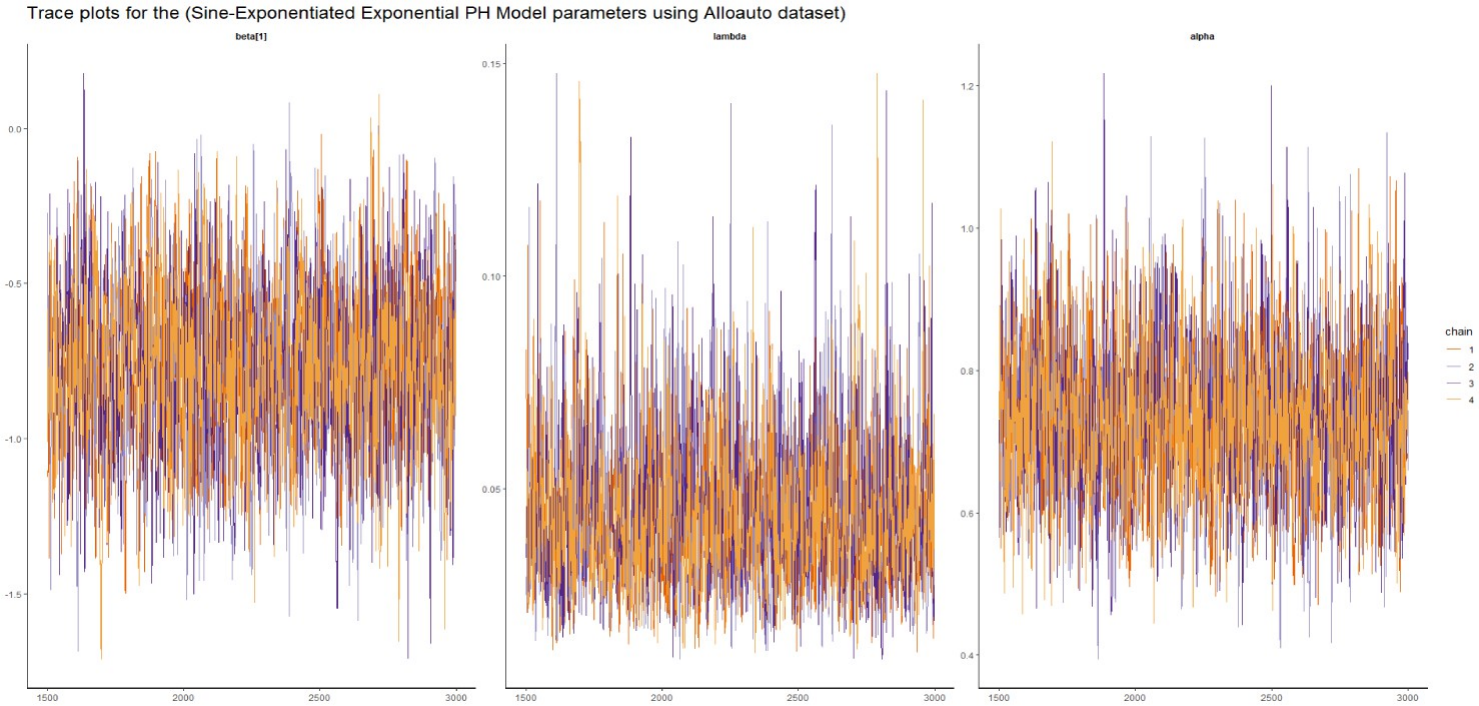
The trace plots of the posterior parameters for the SEE-PH model using Alloauto data.

Here we presented the trace plot for all the competitive models of our study as shown in Figs 14–17.

#### 5.2.3 Model comparison for Dataset II

For the SW-PH model, the WAIC and LOOIC values are both 451.3. The SL-PH model has a slightly lower WAIC and LOOIC of 450.60. The SG-PH model has a higher WAIC and LOOIC of 481.70, while the SEE-PH model has a WAIC and LOOIC of 472.30.

Based on this comparison, the SL-PH model appears to have the best fit to the Alloauto dataset, as it has the lowest WAIC and LOOIC values among the models evaluated. For the SW-PH model, the WAIC and LOOIC values are both 451.3. The SL-PH model has a slightly lower WAIC and LOOIC of 450.60. The SG-PH model has a higher WAIC and LOOIC of 481.70, while the SEE-PH model has a WAIC and LOOIC of 472.30.

Based on this comparison, the SL-PH model appears to have the best fit to the Alloauto dataset, as it has the lowest WAIC and LOOIC values among the models evaluated as presented in Table 4.

**Table 4:** Bayesian model comparison for the SW-PH, SL-PH, SG-PH, and SEE-PH models using Alloauto dataset.

Model	WAIC	LOOIC
SW-PH	451.3	451.3
SL-PH	450.60	450.60
SG-PH	481.70	481.70
SEE-PH	472.30	472.30

## 6 Discussions and conclusions

Our primary objective in this study was to develop and evaluate a Bayesian survival modeling approach for PH regression models. Specifically, we focused on utilizing the Sine-G family of distributions as a baseline hazard family.

By incorporating the Sine-G family, which includes distributions such as the Weibull, exponentiated exponential, Lomax, and Gompertz distributions, we aimed to enhance the modeling capabilities of our approach. These distributions offer a flexible range of hazard forms, allowing us to capture various patterns observed in survival data.

Through rigorous evaluation and analysis, we compared the performance of different baseline hazards within the Sine-G family. Our goal was to identify the most suitable choice for our proposed Bayesian survival modeling approach.

Based on our findings, we determined that the SL-PH model demonstrated superior performance compared to the other baseline hazards considered. This model exhibited greater flexibility and accuracy in representing the HRF within proportional hazard regression models.

By utilizing the SL-PH model, we were able to effectively capture a wide range of hazard shapes, including increasing, decreasing, and bathtub-shaped hazards. This flexibility enabled a more comprehensive understanding of the underlying survival patterns and improved the accuracy of our predictions.

To implement our proposed approach, we used the R programming language and the STAN probabilistic programming framework. This combination ensured efficient computation and reliable inference of model parameters, thereby enhancing the reliability and applicability of our findings.

In summary, our study successfully developed and evaluated a Bayesian survival modeling approach for PH regression models using the Sine-G family of distributions as baseline hazards. The SL-PH model emerged as the preferred choice due to its superior flexibility and accuracy in representing hazard forms. Our findings contribute to advancing the field of survival analysis and provide a valuable tool for researchers and practitioners working with PH regression models in medical research and clinical practice.

## 7 Future work

In summary, future work in this area could involve exploring additional distributions within the Sine-G family, incorporating covariates into the proportional hazard regression models, comparing alternative approaches, validating the proposed approach using cross-validation or external datasets, and applying the methodology to other disease domains. These efforts will further enhance the flexibility, predictive power, and generalizability of Bayesian survival modeling, making it a valuable tool for analyzing time-to-event data in various medical research and clinical settings.

In summary, our future work will involve developing and extending the trigonometric probability distributions to parametric survival regression models, cure models, competing risk models, frailty models, multistate models, longitudinal and joint models, as well as spatial parametric survival models. We will incorporate covariates into the modeling framework, conduct comparative studies, validate the methodology using cross-validation or external datasets, and apply it to other disease domains. These efforts will further enhance the flexibility, predictive power, and generalizability of Bayesian survival modeling, making it a valuable tool for analyzing time-to-event data in various medical research and clinical settings.

## Supporting information

S1 File (DOCX)

## Abbreviations

The following abbreviations are used in this manuscript:

CDF: cumulative density function
CHRF: cumulative hazard rate function
ELPD: expected log predictive density
HMC: Hamiltonian Monte Carlo
HRF: hazard rate function
LOOIC: leave-one-out information criterion
PDF: probability density function
PH: proportional hazard
SEE-PH: sine-exponentiated exponential proportional hazard
SEE: sine-exponentiated exponential
SF: survival function
SG-PH: sine-Gompertz proportional hazard
SG: sine-Gompertz
SL-PH: sine-Lomax proportional hazard
SL: sine-Lomax
SW-PH: sine-Weibull proportional hazard
SW: sine-Weibull
WAIC: Watanabe Akaike information criterion
