# Supplementary material for: Sine-G family of distributions in Bayesian survival modeling: A baseline hazard approach for proportional hazard regression with application to right-censored oncology datasets using R and STAN
Source: PLoS One. 2025 Mar 13;20(3):e0307410. doi: 10.1371/journal.pone.0307410 (PMC11906061; doi:10.1371/journal.pone.0307410)
Supplement: S1 File — (DOCX) [file pone.0307410.s001.docx]

For reproducibility purposes, we have published the code and analysis for our study on an Rpubs page. We encourage readers to visit the https://rpubs.com/Geesaale/1116454, and https://rpubs.com/Geesaale/1116455, section of our publication, where they will find a link to the Rpubs page containing the complete R code. By accessing the Rpubs page, readers will have the opportunity to review the code, scripts, and functions used in our analysis. This transparency allows for a thorough understanding of our methodology and enables readers to replicate the study for validation and further exploration.
